# Stimulus-Specific Expression, Selective Generation and Novel Function of Grass Carp (*Ctenopharyngodon idella*) IL-12 Isoforms: New Insights Into the Heterodimeric Cytokines in Teleosts

**DOI:** 10.3389/fimmu.2021.734535

**Published:** 2021-09-16

**Authors:** Xingyang Qiu, Hao Sun, Dan Wang, Jingqi Ren, Xinyan Wang, Anying Zhang, Kun Yang, Hong Zhou

**Affiliations:** School of Life Science and Technology, University of Electronic Science and Technology of China, Chengdu, China

**Keywords:** grass carp, Il-12, p35 paralogues, heterodimeric form, Th17-like response

## Abstract

Interleukin-12 (IL-12) is a heterodimeric cytokine composed of a p35 subunit specific to IL-12 and a p40 subunit shared with IL-23. In this study, we unveiled the existence of two *p35* paralogues in grass carp (named *gcp35a* and *gcp35b*). Notably, *gcp35a* and *gcp35b* displayed distinct inducible expression patterns, as poly I:C merely induced the gene expression of *gcp35a* but not *gcp35b*, while recombinant grass carp interferon-gamma (rgcIfn-γ) only enhanced the transcription of *gcp35b* but not *gcp35a*. Moreover, the signaling mechanisms responsible for the inducible expression of *gcp35a* and *gcp35b* mRNA were elucidated. Because of the existence of three grass carp *p40* genes (*gcp40a, gcp40b* and *gcp40c*) and two *p35* paralogues, six gcIl-12 isoforms were predicted by 3D modeling. Results showed that gcp40a and gcp40b but not gcp40c had the potential for forming heterodimers with both gcp35 paralogues *via* the disulfide bonds. Non-reducing electrophoresis experiments further disclosed that only gcp40b but not gcp40a or gcp40c could form heterodimers with gcp35 to produce secretory heterodimeric gcp35a/gcp40b (gcIl-12AB) and gcp35b/gcp40b (gcIl-12BB), which prompted us to prepare their recombinant proteins. These two recombinant proteins exhibited their extensive regulation on Ifn-γ production in various immune cells. Intriguingly, both gcIl-12 isoforms significantly enhanced the transcription of *il-17a/f1* and *il-22* in lymphocytes, and their regulation on *il-17a/f1* expression was mediated by Stat3/Rorγt signaling, supporting the potential of gcIl-12 isoforms for inducing Th17-like responses. Additionally, stimulatory effects of gcIl-12 isoforms on *il-17a/f1* and *ifn-γ* expression were attenuated by gcTgf-β1 *via* suppressing the activation of Stat3 signaling, implying that their signaling could be manipulated. In brief, our works provide new insights into the inducible expression pattern, heterodimeric generation and functional novelty of Il-12 isoforms in teleosts.

## Introduction

Interleukin-12 (IL-12) is a heterodimeric cytokine composed of two subunits, p35 and p40 covalently bound through an inter-chain disulfide bond (1). The co-expression of two subunits in the same cells is vital to the formation of IL-12, although the expression of each subunit is differently regulated (2). Since p35 subunit is specific to IL-12 and p40 subunit is shared with IL-23 (3), the production of IL-12 heterodimer is limited by p35 expression (4). In mammals, IL-12 acts on the target cells by binding to heterodimer receptors IL-12Rβ1 and IL-12Rβ2 (5), and activates the tyrosine kinase 2 (TYK2) and Janus kinase 2 (JAK2), respectively (6). Activation of JAK2 predominantly results in signal transducer and activator of transcription 4 (STAT4) phosphorylation and ultimately leads to interferon-gamma (IFN-γ) production (7) as well as Th1 cell differentiation (8). Besides STAT4, STAT3 also participates in the regulation of IL-12 on Th1 differentiation (9).

Unlike in mammals, multiple *p35* and *p40* paralogues have arisen in teleosts due to the teleost-specific whole-genome duplication (WGD) events (10). In various fish species, *p35* paralogue gene expressions are induced by poly I:C, LPS, Il-6, Il-1β, Ifn-γ (11), *Nocardia seriolae* (12), and *Yersinia ruckeri* (13), while *p40* paralogue transcriptions are stimulated by immune stimuli like Il-1β, Tnf-α3 (11), viral hemorrhagic septicemia virus (VHSV) and *Yersinia ruckeri* (14). However, the exact mechanisms governing the transcription of these fish *p35* paralogues remain unclear. Notably, the existence of multiple *p35* and *p40* paralogues may lead to multiple Il-12 isoforms in fish. Accordingly, recombinant Il-12 isoforms of two teleosts, amberjack (12) and rainbow trout (14) have been prepared, and recombinant proteins had classical regulatory effects on the expression of *ifn-γ*. It is noteworthy that rainbow trout Il-12 isoforms possess distinct activities in the induction of *il-10* gene expression (14). However, the roles of these distinct *p35* and *p40* paralogues in generating Il-12 isoforms are unknown, and at least two issues are needed to be addressed in fish species: Firstly, whether the inducible expression of *p35* paralogues is stimuli-specific; Secondly, whether all heterodimer combinations of *p35* and *p40* paralogues exist.

In this study, we isolated and identified a new *p35* gene in grass carp, which was named *gcp35b* differing from the *gcp35a* reported previously (15). Subsequently, immune stimuli-regulated expression profiles of two *gcp35* paralogues and the related signaling mechanisms were examined in grass carp monocytes/macrophages, providing information on the specific expression patterns of two *gcp35* paralogues. It is known that there are three grass carp *p40* genes (*gcp40a, gcp40b* and *gcp40c*) (16), raising a question about how many Il-12 isoforms exist in grass carp. Along this line, the six predicted gcIl-12 heterodimers were constructed by 3D modeling and the existence of secretory gcIl-12 isoforms was identified by non-reducing electrophoresis, supporting that only two heterodimeric Il-12 isoforms were presented in grass carp. To better understand the function of two gcIl-12 isoforms, their recombinant proteins were prepared and their potentials to stimulate Ifn-γ production were confirmed in various immune cells. Unexpectedly, the gcIl-12 isoforms exhibited the ability to function as novel regulators of Th17-like response in grass carp lymphocytes. Finally, inhibitory effects of Tgf-β1 on gcIl-12 isoform signaling were elucidated in the same cell model.

## Materials and Methods

### Animal and Reagents

Healthy grass carp weighing about 0.75-1.00 kg was obtained from Chengdu Tongwei Aquatic Science and Technology Company (Chengdu, China). After an adaptation period of 7 days, the fish was anaesthetized in 0.05% MS222 (Sigma-Aldrich, MO, USA) and sacrificed. The head kidney was taken from the fish for head-kidney leukocytes (HKLs), lymphocytes and monocytes/macrophages isolation. All animal experiments were reviewed and conducted according to the Regulation of Animal Use in Sichuan province, China, and were approved by the ethics committee of the University of Electronic Science and Technology of China.

The recombinant gcIfn-γ (rgcIfn-γ, 500 ng/mL) (17), rgcTgf-β1 (100 ng/mL) and anti-gcTgf-β1 mAb (1:2000 diluted) were prepared referring to our previous studies (18, 19). LPS from *Escherichia coli* O55:B5 and poly I:C were purchased from Sigma Aldrich, and their doses used in this study were determined according to our previous studies (17, 18, 20, 21). Inhibitors for NF-κB (PDTC, 0.25 µM, Sigma Aldrich, St. Louis, USA), ERK1/2 (PD98059, 30 μM, Merck, Bad Soden, Germany), JNK (SP600125, 6 μM, Merck), P38 (SB202190, 30 µM, Merck), activin receptor-like kinase 5 (ALK5, TGF-β1 RI Kinase inhibitor VIII, 2 µM, Calbiochem, EMD Chemicals, San Diego, USA), RORγt (SR1001, 15 μM, Sigma-Aldrich) and STAT3 (STAT3 VI, 30 μM, Sigma-Aldrich) were used and an equal amount of solvent was used in the control groups in the experiments. The use of these inhibitors referred to previous studies (18, 22–25). Among them, anti-gcTgf-β1 mAb and ALK5 inhibitor were used to confirm the role of Tgf-β1 signaling in limiting rgcIl-12BB actions.

### Molecular Cloning of gcp35b cDNA and Sequence Analysis of p35 Homologues

Total RNA was extracted from the grass carp head kidney with TriPure Isolation Reagent (Roche, Basel, Switzerland) and then reverse transcribed to cDNA by using M-MLV reverse transcriptase (Promega, Madison, USA) with oligo d(T)_18_ as the primer. The potential sequence encoding *gcp35b* was obtained by searching the grass carp genome sequence (http://www.ncgr.ac.cn/grasscarp/) with the offline BLAST tool based on the sequence of zebrafish *p35b* (GenBank ID: XM_017352586). The full-length *gcp35b* cDNA sequence was amplified from the grass carp head kidney cDNA by Phusion High-Fidelity DNA Polymerase (Vazyme, Nanjing, China) and sequenced. The cDNA and deduced amino acid sequence of *gcp35b* were analyzed by using the ExPASy Molecular Biology server (http://www.expasy.org). The molecular weight and isoelectric point of the putative gcp35b were predicted by Compute PI/Mw tool (http://web.expasy.org/compute pi/) and the deduced signal peptide was predicted using SignalP (http://www.cbs.dtu.dk/services/SignalP/). The potential N-glycosylation sites of grass carp Il-12 subunits were predicted by NetNGlyc 1.0 Server (http://www.cbs.dtu.dk/services/NetNGlyc/). Gene synteny of the *p35* loci was analyzed using the data from the NCBI database (https://www.ncbi.nlm.nih.gov/). The multiple amino acid alignments and the phylogenetic tree were constructed by MEGA7.1 software (https://www.megasoftware.net/).

### Structural Modeling of Grass Carp Il-12 Heterodimer

The structural models of grass carp Il-12 heterodimer were constructed by SWISS-MODEL (http://swissmodel.expasy.org) based on the human Il-12 crystal structure (PDB:1F45). The molecular graphics visualization tool RasMol 2.7.2.1 (http://www.openrasmol.org/) was used to display the structural models. These predicted models were evaluated by the Qualitative Mean Energy Analysis Distance Constraint Global (QMEANDis Co Global) of SWISS-MODEL (https://swissmodel.expasy.org/qmean/).

### Plasmid Construction

To study the existence of gcIl-12 isoforms, the coding sequences (CDS) of grass carp *p35a* and *p35b* were amplified by Phusion High-Fidelity DNA Polymerase (Vazyme) with the primers listed in **Supplementary Table 1**. The CDS of *gcp35a* and *gcp35b* were separately subcloned into p3×FLAG-CMV-7.1 (Promega) to obtain the N-terminal FLAG-tagged gcp35a (gcp35a-FLAG) and gcp35b (gcp35b-FLAG) plasmids. The C-terminal HIS-tagged gcp40a/b/c (gcp40a/b/c-HIS) expression plasmids have been used in our previous study in which gcp19 and gcp40a/b/c heterodimeric assembly is investigated (16). The integrity of the inserted DNA fragments was verified by sequencing. The highly purified and endotoxin-free DNA plasmids were extracted from *Escherichia coli* (*E. coli*) by using a TIAN prep Mini Plasmid Kit (Tiangen, Beijing, China) for subsequent transfection. The HEK293 cells (2 × 10^4^ cells/35 mm culture dish) were transiently transfected with different combinations of gcp35a/b-FLAG and gcp40a/b/c-HIS plasmids (1.2 µg of each plasmid/35 mm culture dish) by using Lipofectamine 2000 Reagent (Thermo Scientific, Carlsbad, USA), separately. Forty-eight hours after transfection, the cell culture supernatants were collected and examined by a non-reducing Western Blotting (WB) assay.

### Recombinant Expression and Purification of gcIl-12 Isoforms

To acquire the recombinant gcIl-12 isoforms, a (GGGGS)_3_ linker was used to link the p35 subunit and p40 subunit (p40-(GGGGS)_3_-p35). Briefly, the cDNA sequences encoding mature gcp35a/b were amplified by primers of *p35a/b*-*Hind*III G4S3 linker F, *p35a*-C-myc+6*his TGA *Xho*I R and *p35b*-*Xho*I R (**Supplementary Table 1**) with Phusion High-Fidelity DNA Polymerase (Vazyme) and then cloned into dhfr-deficient CHO (CHO-dhfr^-^/^-^) cell expression vector pSV2-dhfr (Youbio, Changsha, China) after digested with *Hind*III and *Xho*I (NEB). Next, the DNA fragment encoding the gcp40b was amplified by PCR using the primers with *Hind*III restriction site (**Supplementary Table 1**) and subsequently inserted into the expression vector containing *gcp35a* or *gcp35b* after the digestion with *Hind*III. The integrity of the inserted DNA fragments was verified by sequencing. Then the highly purified and endotoxin-free DNA plasmids were extracted from *E. coli* by using a TIAN prep Mini Plasmid Kit (Tiangen).

CHO cells were seeded at a density of 2 × 10^4^ cells/35 mm culture dish in IMDM medium (Gibco, NY, USA) supplemented with 10% FBS (Gibco), 1% HT (Gibco) and 1% antibiotic-antimycotic (Thermo Scientific) for 24 h before transfection. The plasmids for two gcIl-12 isoforms were separately transfected into the CHO cells by Lipofectamine 2000 according to the manufacturer’s instructions (Thermo Scientific). Forty-eight hours after transfection, cells were selected in the IMDM medium with 10% FBS (Gibco) and 1% antibiotic-antimycotic (Thermo Scientific) which contains different concentrations of methotrexate (MTX) (Life Technologies, Gaithersburg, MD). MTX is commonly used as a selective antibiotic in the dihydrofolate reductase (dhfr) selection system. After cultured in high dose of MTX (500 nM), the transformed clones were isolated using 10 µL pipette tips and screened by WB assay. The selected clones were cultured in 5 mL SFM4CHO medium (Gibco) in 50 mL BD tubes at 37°C with shaking at 180 rpm for 48 h and then those cells were transferred to a 125 mL flask and cultured with 30 mL SFM4CHO medium at 32°C with shaking at 180 rpm for a week. The rgcIl-12 isoforms in the culture medium were purified by His Trap affinity column (GE Healthcare, Waukesha, USA) and desalted by the Superdex-G25 prep grade column (GE Healthcare). The molecular weight and purity of purified proteins were analyzed on SDS-PAGE and WB. Finally, the rgcIl-12 isoforms were lyophilized and then stored at −80°C for further use.

### SDS-PAGE and WB Assay

The non-reducing electrophoresis with SDS but without β-mercaptoethanol (β-ME) was used to determine the gcIl-12 heterodimer composition. In this scenario, the cell culture media of HEK293 cells transfected with gcp35a/b and gcp40a/b/c expression plasmids were harvested at 48 h after transfection and added a 5×loading buffer without β-ME, and then the samples were boiled at 70°C for 10 min. After that, these samples were separated on 10% SDS-PAGE, and then electrophoretically transferred to a PVDF membrane (Millipore, Billerica, MA). The membrane was blocked by TBST buffer (1% Tween) containing 10% (wt/vol) defatted dry milk for 2 h at room temperature and then incubated with anti-HIS mAb (1:600, ZSGB-BIO, Beijing, China) or anti-FLAG mAb (1:5000, Cell Signaling Technology, MA, USA) overnight at 4°C. The membrane was exposed to horseradish peroxidase (HRP)-conjugated goat anti-rabbit secondary antibody (1:5000, ZSGB-BIO) for 2 h at room temperature. Finally, signals were detected using an ECL kit (Roche Diagnostics, Mannheim, Germany) according to the manufacturer’s instructions.

To detect the activation of signaling pathways, the cell lysates were separated on 12% SDS-PAGE and the signaling molecules were detected by WB assay by using specific primary antibodies for phosphorylated ERK (anti-pERK1/2, 1:1000, Cell Signaling Technology), JNK (anti-pJNK, 1:1000, Cell Signaling Technology), p38 (anti-p-p38, 1:1000, Cell Signaling Technology), IκBa (anti-p-IκBa, 1:1000, Cell Signaling Technology), STAT3 (anti-pSTAT3, 1:1000, Anaspec, Fremont, CA, USA), and the β-actin (anti-β-actin 1:5000, Boster, Wuhan, China) as the loading control. These antibodies detecting the activated state of signaling molecules are raised against peptides based on the phosphorylation sites of each signaling molecule (**Figure S5B**), and they have been applied and effectively recognized the corresponding molecules in grass carp and other fish species (23, 26–28). The predicted sizes and amino acid sequence alignment analysis of these signaling molecules in grass carp are shown in **Figure S5**.

The molecular weight and purity of two rgcIl-12 isoforms were evaluated by SDS-PAGE and verified by Western blotting using anti-gcp35a pAb (1:1000), anti-gcp35b pAb (1:1000) and anti-gcp40b pAb (1:1000, Biogot Technology, Nanjing, China). In the experiments, anti-gcp35a pAb and anti-gcp35b pAb were custom products from Abmart Inc. (Shanghai, China). To validate anti-gcp35a pAb specificity, the recombinant gcp35a and grass carp HKLs lysates were analyzed by WB in which the membrane was incubated with anti-gcp35a pAb (1:1000) or the antibody pre-absorbed with rgcp35a (**Figures S6A, B**). Following the same procedures, the lysates of grass carp HKLs treated with or without heat-killed *Aeromonas hydrophila* (*A. hydrophila*) [MOI 1:1, which has been described previously (29)] for 6 h were used to verify the specificity of the gcp35b antibody (**Figures S6C, D**). Additionally, the specificity of anti-gcp40b pAb has been demonstrated and used in previous research (30).

### Isolation and Culture of Grass Carp HKLs, Lymphocytes and Monocytes/Macrophages

Grass carp HKLs were prepared by discontinuous density gradient centrifugation with Ficoll-Hypaque (1.083 kg/L, TBD science, Tianjin, China) referring to our previous studies (23). Briefly, head kidney was obtained from freshly sacrificed fish and then squeezed to release the cells. After the tissue debris was removed, the cells were layered on Ficoll-Hypaque and centrifuged at 1580 × *g* for 30 min at 20°C. After centrifugation, the leukocytes at the interface were collected and washed twice with PBS. The cells were resuspended in RPMI-1640 medium (Gibco) with 10% FBS (Gibco) and 1% antibiotic-antimycotic (Thermo Scientific). About 6 × 10^5^ cells/well were seeded in a 24-well plate (Nunc-Intermed, Roskilde, Denmark) and incubation overnight at 26°C under 5% CO_2_ and saturated humidity. For lymphocytes and monocytes/macrophages isolation, the cell suspension was centrifuged at 400 × *g* for 25 min in a density gradient column formed by two solutions with different densities from fish lymphocyte and monocyte preparation kit (TBD, Tianjin, China) according to the previous study (17). About 6 × 10^5^ cells/well lymphocytes were seeded in a 24-well plate and incubated overnight at 26°C under 5% CO_2_ and saturated humidity. The collected monocytes/macrophages were resuspended in RPMI-1640 medium (Gibco) with 1% FBS (Gibco) and 1% antibiotic-antimycotic (Thermo Scientific) and seeded in a 24-well plate with 5 × 10^6^ cells/well. After 2 hours of incubation, the unattached cells on the plate were washed away with PBS. Then the cells were cultured in RPMI-1640 medium (Gibco) with 10% FBS (Gibco) and 1% antibiotic-antimycotic (Thermo Scientific). In the following day, the cells were treated with different drugs in individual experiments.

### Gene Expression Analysis by Real-Time Quantitative PCR (RT-qPCR)

Total RNA was extracted from the cells and then reverse transcribed to cDNA by using M-MLV reverse transcriptase (Promega). The *gcp35a*, *gcp35b*, *ifn-γ*, *il-17a/f1*, *il-22* and *β-actin* mRNA levels were assessed by using RT-qPCR. In brief, RT-qPCR was performed on the Bio-Rad CFX96™. Real-time detection system (Bio-Rad, Hercules, CA) in a total volume of 10 µL with 4 µL of 2.5 × RealMasterMix (Tiangen, Beijing, China), 0.5 µL of 20 × SYBR Green, 1 µL of cDNA, 0.2 µL of each of forward primer and reverse primer and 4.1 µL of deionized water. The amplification program was 94°C for 2 min, followed by 35 cycles of 94°C for 20 s, 54-60°C (54°C for *p35a*, 60°C for *p35b*, 60°C for *ifn-γ*, 60°C for *il-17a/f1*, 60°C for *il-22* and 59°C for *β-actin*) for 20 s and 65°C for 20 s. All PCR products were visualized on a 2% agarose gel to check the PCR amplification. To estimate the amplification efficiency, the standard curve for each target molecule was generated by 10-fold serial dilutions (from 10^-1^ to 10^-6^ fmol/µL) of a plasmid containing the individual target gene sequences as the PCR template. The melting analysis was routinely performed to check the authenticity of the PCR products (31). The relative expression levels of target genes were analyzed using the 2^-ΔΔCt^ method (32) by normalization with *β-actin* gene expression and presented as fold changes compared with the matched controls. The primers for the RT-qPCR are listed in **Supplementary Table 1**.

### Competitive-Inhibition Enzyme Linked Immunosorbent Assay (ELISA)

The grass carp HKLs culture medium was collected to measure the concentration of grass carp Ifn-γ by competitive-inhibition ELISA. In the assay, anti-gcIfn-γ pAb was a custom product from Abmart (Shanghai, China). To validate anti-gcIfn-γ pAb specificity, the recombinant gcIfn-γ (rgcIfn-γ) was used and analyzed by SDS-PAGE (**Figure S10A**) and WB in which the membrane was incubated with anti-gcIfn-γ pAb (1:1000) (**Figure S10B**) or the antibody pre-absorbed with rgcIfn-γ (**Figure S10C**). To establish a competitive-inhibition ELISA, an orthogonal experiment was designed by setting different coating concentrations of gcIfn-γ, and the dilution ratios of primary antibody and secondary antibody. The optimized amount of coating antigen was 100 ng/well of rgcIfn-γ and concentrations of the antibodies were 1:1000 (v/v) of anti-gcIfn-γ pAb (1:1000) and HRP-conjugated goat anti-rabbit secondary antibody. Subsequently, a standard curve was built according to the inhibition ratio and the corresponding protein concentrations (**Figure S11**).

In this experiment, a 96-well polystyrene plate (Costar, Cambridge, MA, USA) was coated with 100 ng/well of rgcIfn-γ at 4°C for 16 h and then blocked with 5% defatted milk plus 0.3% BSA in PBS for 3 h at room temperature. At the same time, 50 µL of culture medium or the titrated rgcIfn-γ and 50 µL of anti-gcIfn-γ pAb (1:1000) (Abmart) were mixed and incubated at room temperature for 2 h. After that, the plate was washed with PBST (0.05% Tween-20 in PBS) for three times and 100 µL of the medium-antibody mixture was added into each well and further incubated at room temperature for 2 h. The plate was washed with PBST for five times, and then 100 µL of HRP-conjugated goat anti-rabbit secondary antibody (1:1000, ZSGB-BIO) was added into each well. After 2 h incubation at room temperature, the plate was washed with PBST for five times and 100 µL of substrate buffer (TMD, Tiangen) was added into the wells and incubated for 20 min at 37°C. The reaction was stopped by 2 M H_2_SO_4_ and the absorbance values were measured at 450 nm with the iMark Microplate Reader (Bio-Rad). Control groups were pre-coated with BSA followed by the same procedures as described above. The concentrations of samples were extrapolated from a standard curve for gcIfn-γ inhibition.

### Glycosylation Analysis of rgcIl-12AB and rgcIl-12BB

To examine if the rgcIl-12AB and rgcIl-12BB expressed by CHO cells were glycosylated, PNGase F (NEB), a glycosidase, was used to digest rgcIl-12AB and rgcIl-12BB following the manufacturer’s instructions. According to the method in a previous study (33), 10 µg of rgcIl-12AB or rgcIl-12BB was denatured in 1 × Glycoprotein Denaturing buffer (0.5% SDS, 40 mM DTT) for 10 min in boiling water, and added to 10 × G2 Reaction buffer (500 mM PBS, pH 7.5) containing 2 µL of 10% NP-40 and 2 µL of PNGase F. The mixture was incubated at 37°C for 2 h and the samples were assayed by SDS-PAGE and WB.

### Data Interpretation and Statistical Analysis

Data were collected from at least three independent experiments and all results were expressed as mean ± SEM with four independent replicates (N = 4). GraphPad Prism 7 software (GraphPad Inc., San Diego, CA, USA) was used to test the normality and homogeneity of variance of all data according to the instructions of GraphPad Prism (https://www.graphpad-prism.cn/guides/prism/8/statistics/index.htm), and then perform statistical analyses. For comparison between two groups, Student’s *t*-test was performed. Multiple group comparison was conducted by one-way ANOVA followed by a Tukey’s multiple comparisons test. Significant differences and highly significant differences were considered at *p* < 0.05 and *p* < 0.01, respectively.

## Results

### Molecular Cloning of gcp35b and Sequence Analysis of p35 Homologues

**Figure 1:**
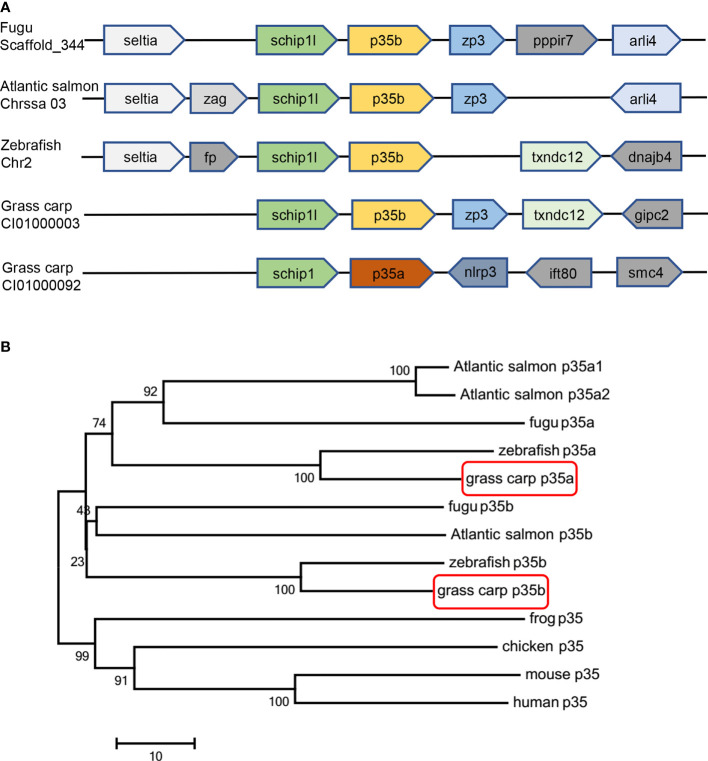
Gene synteny and phylogenetic tree analysis of *p35* homologues. **(A)** Gene synteny analysis of grass carp *p35a*, grass carp *p35b*, fugu *p35b*, zebrafish *p35b*, and Atlantic salmon *p35b*. The direction of the box arrow indicated gene transcription orientation. **(B)** An unrooted phylogenetic analysis of *p35* amino acid sequences in various vertebrates. The tree was constructed by the neighbor joining method by using MEGA 7 software. The numbers indicate the bootstrap confidence values obtained for each node after 1000 replications. The GenBank accession numbers of *p35* are as follows: human (NM_000882), mouse (NM_008351), chicken (NM_213588), frog (XM_012963291), fugu *p35a* (H2SI185), fugu *p35b* (NM_001078598), Atlantic salmon *p35a1* (HG917954.1), Atlantic salmon *p35a2* (HG917955.1), Atlantic salmon *p35b* (HG917956.1), zebrafish *p35a* (NM_001007107), zebrafish *p35b* (XM_017352586), grass carp *p35a* (KF944667) and grass carp *p35b* (MZ393470).

Although two or three *p35* paralogues were found in a variety of teleosts, only one *p35* gene has been reported so far in grass carp (nominated as *gcp35a* in the present study). In this study, another grass carp *p35* cDNA (named *gcp35b*) was isolated, which contains 582 bp nucleotides encoding a 193-aa polypeptide (**Figure S1**). Moreover, *gcp35b* loci exhibited a conserved synteny with its homologues in other teleost species like fugu, Atlantic salmon and zebrafish, and both *gcp35* paralogues were in proximity with the conserved gene *schip1* (**Figure 1A**). Subsequent phylogenetic analysis of *p35* showed three sub-clades: teleost *p35a*, *p35b* branches, and the branch consisted of frog, chicken, mouse and human *p35* genes (**Figure 1B**). The multiple amino acid sequences alignment of *gcp35b* with its homologues showed that it had the highest identity with zebrafish *p35b* (50.93%) and shared low identities with other homologues in teleosts (fugu *p35a* 17.47%, fugu p35b 19.70%, zebrafish *p35a* 18.22% and grass carp *p35a* 20.07%). In addition, *gcp35b* also shared lower identities (14.87%, 12.64%, 14.50% and 13.01%) with the human, mouse, chicken and frog *p35* homologues (**Figure S2A**). Furthermore, the cysteine residues (C^28^, C^60^, C^77^, C^87^, C^100^, C^138^ and C^168^ of gcp35a, C^41^, C^67^, C^84^, C^91^, C^104^, C^142^ and C^170^ of gcp35b) were conserved with p35 homologues in other vertebrate species (**Figure S2A**). The cysteine residues (C^87^ of gcp35a and C^91^ of gcp35b) that form the inter-chain disulfide bond were marked with a star (**Figure S2A**). The glycosylation sites of gcp35a/b were highlighted in **Figure S3**.

### Stimulus-Specific Inductive Expression Patterns of gcp35a and gcp35b in Grass Carp Monocytes/Macrophages

**Figure 2:**
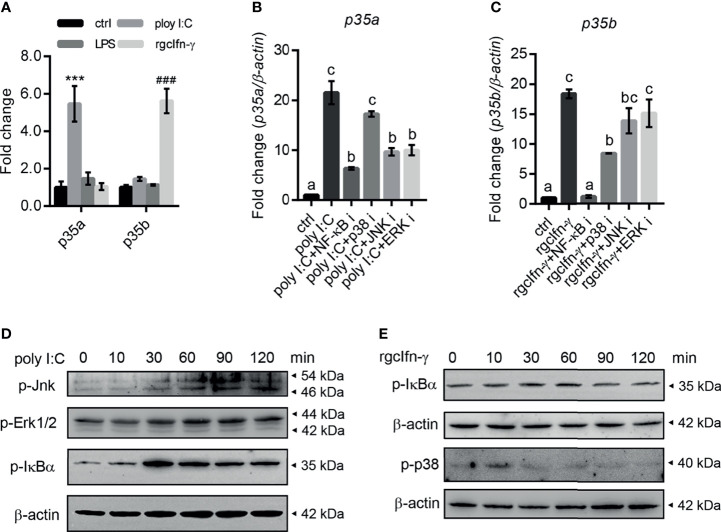
Inductive expression of *gcp35a* and *gcp35b* transcripts after different immune challenges in grass carp monocytes/macrophages. **(A)** The monocytes/macrophages were treated with poly I:C (50 µg/mL), LPS (30 µg/mL) and rgcIfn-γ (500 ng/mL) for 6 h. ***p < 0.001 (*vs.* p35a ctrl), ^###^*p* < 0.001 (*vs.* p35b ctrl). Data are shown as mean ± SEM (N = 4). **(B, C)** The inhibitors for p38 (SB202190, 30 µM), JNK (SP600125, 6 µM), ERK (PD98059, 30 µM) and NF-κB (PDTC, 0.25 µM) were added with poly I:C (50 g/mL), or rgcIfn-γ (500 ng/mL) for 12 h, separately. The *gcp35a* and *gcp35b* mRNA was normalized relative to *β-actin* and expressed as fold changes compared with the control group. Different letters indicate significant differences at *p* < 0.05. Data are shown as mean ± SEM (N = 4). Activation of Jnk, Erk1/2 and IκBα by poly I:C (50 µg/mL) **(D)** and IκBα and p38 by rgcIfn-γ (500 ng/mL) **(E)** for 0, 10, 30, 60, 90 and 120 min in grass carp monocytes/macrophages.

In grass carp monocytes/macrophages, poly I:C (50 µg/mL) significantly up-regulated the mRNA expression of *gcp35a* but not *gcp35b*, while rgcIfn-γ (500 ng/mL) induced the transcription of *gcp35b* but not *gcp35a* (**Figure 2A**). Meanwhile, LPS (30 µg/mL) did not affect the transcript levels of two *gcp35* paralogues (**Figure 2A**). The time course experiments showed that a 12-h treatment with poly I:C and rgcIfn-γ was sufficient to induce a marked expression of *gcp35a* and *gcp35b* (**Figures S4A, B**), and this time point was chosen for later studies. Subsequently, the signaling mechanisms responsible for the regulation of poly I:C and rgcIfn-γ on *gcp35a* and *gcp35b* transcription were elucidated, respectively. The poly I:C-induced mRNA expression of *gcp35a* was partially attenuated by NF-κB, JNK and ERK inhibitors (**Figure 2B**). Meanwhile, the stimulation of rgcIfn-γ on the *gcp35b* mRNA expression was totally blocked by NF-κB inhibitor and partially suppressed by p38 inhibitor (**Figure 2C**). Moreover, poly I:C was effective in stimulating the phosphorylation of Jnk, Erk and IκBα from 30 to 120 min (**Figure 2D**). Meanwhile, rgcIfn-γ significantly induced the phosphorylation of p38 at 10 min and showed a slight stimulation of IκBα phosphorylation at 30 min (**Figure 2E**). Referring to the information available at Cell Signaling Technology’s website (https://www.cellsignal.com/), the molecular sizes of p-JNK, p-ERK1/2, p-IκBα and p-p38 in grass carp were similar to that in mammals (**Figure S5A**).

### 3D Structural Models of Heterodimers Consisted With gcp35 and gcp40 Paralogues

**Figure 3:**
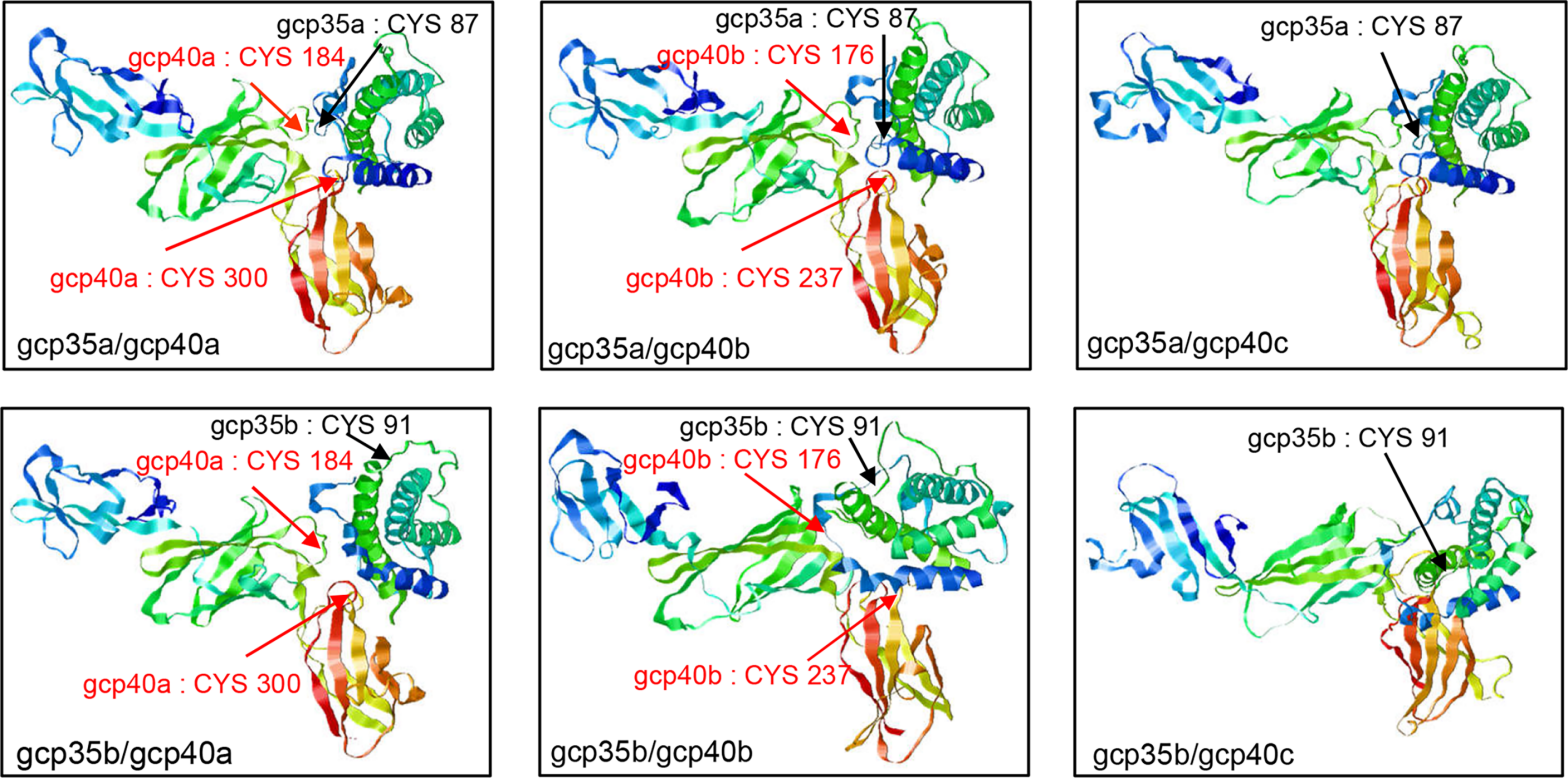
3D structural models of grass carp Il-12 isoforms. The structural models of grass carp Il-12 isoforms were constructed based on the human IL-12 crystal structure (PDB: 1F45). The exposed cysteines at the contact surface of gcp35 paralogues are marked with black arrows and the red arrows indicated the cysteines of gcp40 paralogues.

To learn the structural information of theoretical gcIl-12 heterodimers, their 3D models were constructed based on the human Il-12 crystal structure (PDB: 1F45), thereby revealing the location of those conserved cysteine residues on the contact surface of the predicted heterodimers consisting of gcp35 and gcp40 paralogues (**Figure 3**). The QMEANDisCo Global scores of all gcIl-12 heterodimer 3D models were higher than 0.5 (**Supplementary Table 2**). As shown in **Figure 3**, the conserved cysteine residues on the contact surfaces of the predicted heterodimers included gcp35a (C^87^) and gcp35b (C^91^) marked by black arrows, as well as C^184^ and C^300^ of gcp40a and the C^176^ and C^237^ of gcp40b indicated by red arrows. However, no cysteine residue was found on the contact surface of gcp40c.

### Selective Generation of gcIl-12 Isoforms in HEK293 Cells

**Figure 4:**
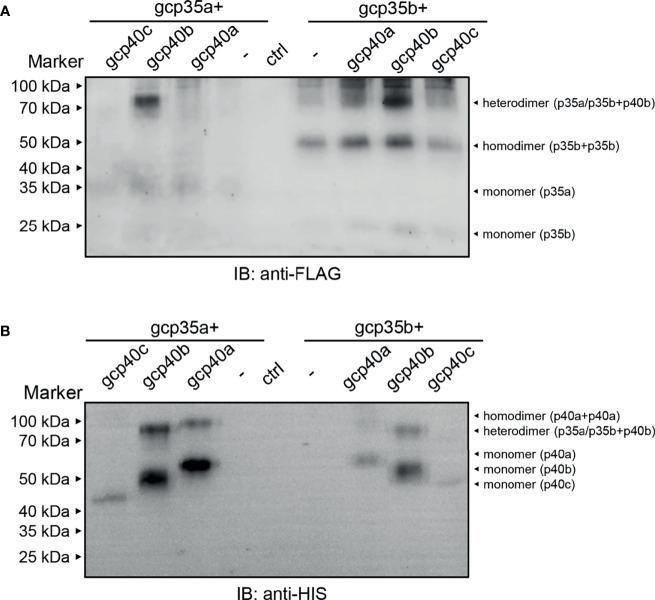
Identification of the grass carp Il-12 heterodimer composition. HEK293 cells were transfected with 1.2 µg of plasmids encoding the individual subunits alone or with a mixture of 1.2 µg each of the two plasmids as indicated in picture. The cell culture media samples were separated under non-reducing conditions (without β-ME). The molecular mass standards (kDa) are indicated. The lane of ctrl means that HEK293 cells were transfected with a mixture of 1.2 µg each of the two vectors [p3×FLAG-CMV-7.1 and pcDNA 3.1/myc-His (–)]. **(A)** Non-reducing WB assay by using anti-FLAG antibody. **(B)** Non-reducing WB assay by using anti-HIS antibody.

To clarify the composition of the gcIl-12 heterodimer, different combinations of gcp35a/b-FLAG and gcp40a/b/c-HIS were overexpressed in HEK293 cells, and then the cell culture media were collected and analyzed under non-reducing conditions. As shown in **Figure 4**, anti-FLAG antibody and anti-HIS antibody could detect the monomeric gcp35a/b (35 kDa and 25 kDa in **Figure 4A**) and gcp40a/b/c (55 kDa, 50 kDa and 43 kDa in **Figure 4B**) showing weak and clear bands, respectively. Notably, anti-FLAG antibody recognized an obvious band with the MW of 75 kDa in overexpressing gcp35a/gcp40b and gcp35b/gcp40b lanes (indicated by “heterodimer”, **Figure 4A**). Consistently, this band was also detected by anti-HIS antibody in the same lanes (indicated by “heterodimer”, **Figure 4B**). In addition, homodimeric gcp35b (50 kDa) was detected by anti-FLAG antibody (right panel, **Figure 4A**). Similarly, homodimeric gcp40a (more than 100 kDa) was detected by anti-HIS antibody (**Figure 4B**).

### Recombinant Expression and Purification of gcIl-12 Isoforms

To explore the biological activity of two main gcIl-12 isoforms as described above, the recombinant gcIl-12AB (rgcIl-12AB) and gcIl-12BB (rgcIl-12BB) were prepared by the CHO cell expression system and purified by His Trap affinity column, and their purity was evaluated by SDS-PAGE and WB. SDS-PAGE analysis showed that the purified rgcIl-12AB (**Figure S7A**) and rgcIl-12BB (**Figure S7D**) were visualized as a single band about 75 kDa and 70 kDa, respectively, and they were larger than their predicted MW (rgcIl-12AB with 58 kDa and rgcIl-12BB with 57 kDa). Furthermore, the single band of purified rgcIl-12AB was verified by WB analysis with anti-gcp35a and anti-gcp40b pAb (**Figures S7B, C**), while a single band for rgcIl-12BB was recognized by both anti-gcp35b and anti-gcp40b pAb (**Figures S7E, F**). The glycosylation of rgcIl-12AB and rgcIl-12BB was assessed by glycosidase digestion. SDS-PAGE analysis showed the MWs of rgcIl-12AB and rgcIl-12BB digested with glycosidase were corresponding to their predicted sizes, respectively (**Figures S8A, B**) and these results were further confirmed by WB assay (**Figures S8C, D**).

### Functional Verification of rgcIl-12 Isoforms in Grass Carp HKLs, Lymphocytes and Monocytes/Macrophages

**Figure 5:**
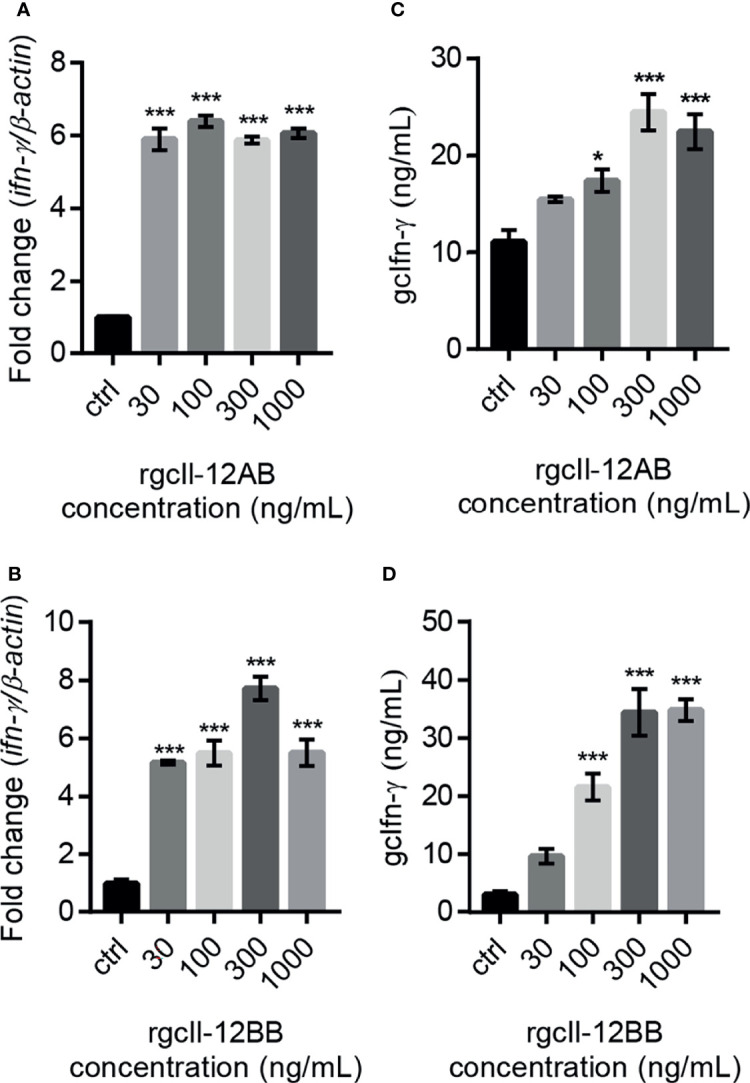
Effects of rgcIl-12 isoforms on Ifn-γ mRNA expression and protein release in grass carp HKLs. HKLs were treated with rgcIl-12AB or rgcIl-12BB (30-1000 ng/mL) for 12 h **(A, B)**. The mRNA levels of *ifn-γ* mRNA were detected by qPCR. The mRNA levels were normalized by *β-actin* and expressed as fold changes compared with the control group. The protein levels of gcIfn-γ in HKLs culture medium were detected by competitive-inhibition ELISA after the HKLs were treated with rgcIl-12AB or rgcIl-12BB (30-1000 ng/mL) for 12 h **(C, D)**. Data are shown as mean ± SEM (N = 4). *p < 0.05 and ***p < 0.001.

**Figure 6:**
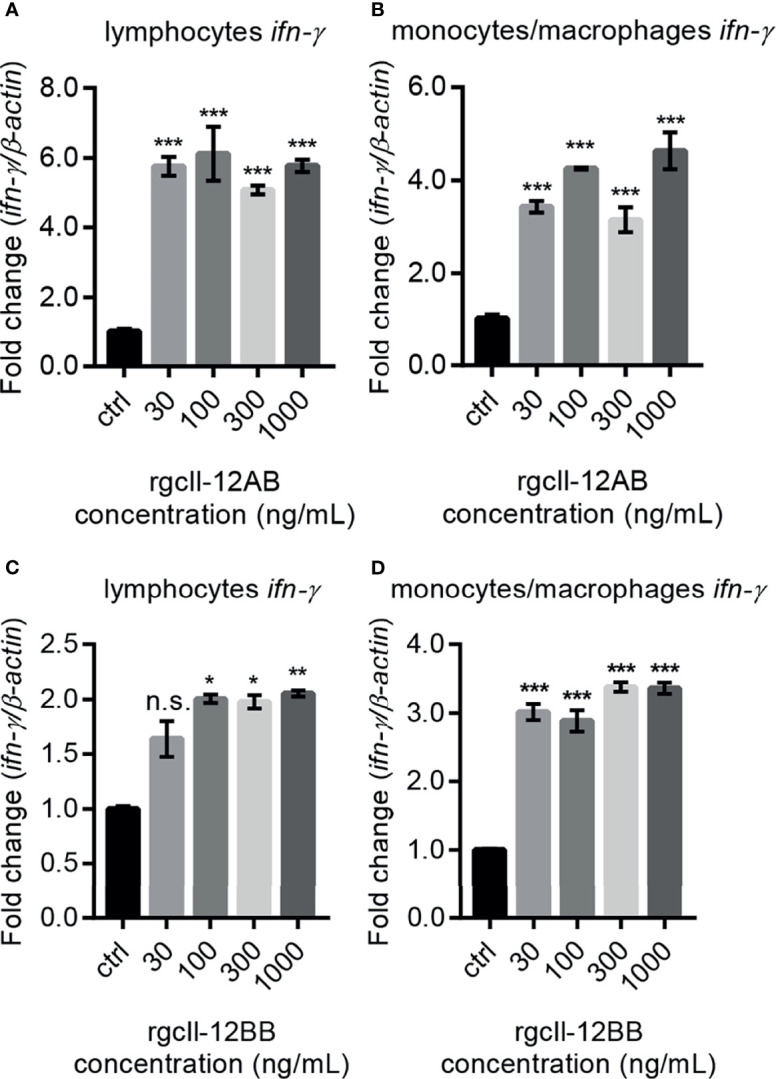
Effects of rgcIl-12 isoforms on *ifn-γ* mRNA expression in grass carp lymphocytes and monocytes/macrophages. Lymphocytes and monocytes/macrophages were treated with 30-1000 ng/mL rgcIl-12AB **(A, B)** or rgcIl-12BB **(C, D)** for 12 h, respectively. The mRNA expression of *ifn-γ* was detected by qPCR and they were normalized by *β-actin* and expressed as fold changes compared with the control group. Data are shown as mean ± SEM (N = 4). *p < 0.05, **p < 0.01, and ***p < 0.001. The “n.s.” indicates no significant.

To investigate the biological activity of two rgcIl-12 isoforms, grass carp HKLs were incubated with different concentrations of rgcIL-12AB or rgcIl-12BB for 12 h. Results showed that rgcIl-12AB (**Figure 5A**) and rgcIl-12BB (**Figure 5B**) could induce *ifn-γ* mRNA expression from 30 to 1000 ng/mL. In parallel, a time course experiment showed that a 12-h treatment of rgcIl-12BB (1000 ng/mL) was sufficient to induce the maximal effect on *ifn-γ* expression in HKLs (**Figure S9**). Furthermore, the competitive-inhibition ELISA results showed that rgcIl-12AB (100-1000 ng/mL) and rgcIl-12BB (100-1000 ng/mL) markedly stimulated the release of Ifn-γ (**Figures 5C, D**). Similarly, in lymphocytes and monocytes/macrophages, a 12-h treatment with rgcIl-12AB or rgcIl-12BB (30-1000 ng/mL) resulted in the increase of *ifn-γ* transcription (**Figures 6A–D**).

### Effects of rgcIl-12 Isoforms on the mRNA Expression of Th17 Related Cytokines in Grass Carp Lymphocytes and Monocytes/Macrophages

**Figure 7:**
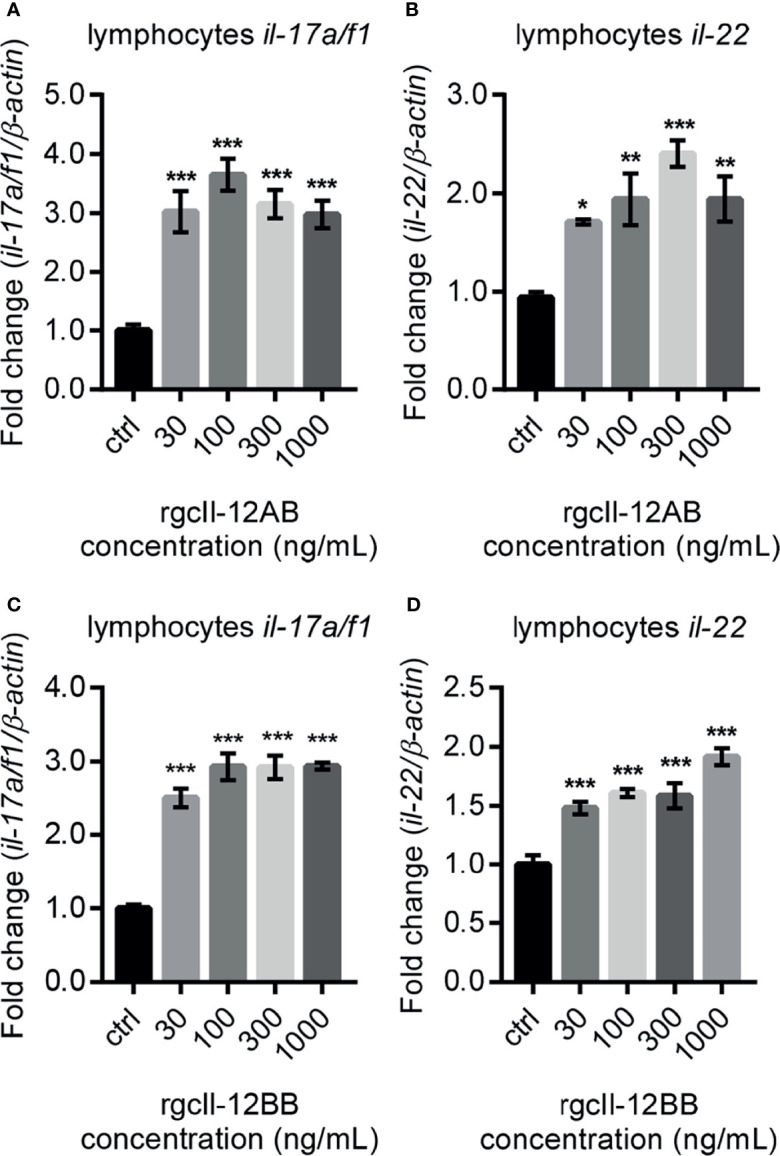
Effects of rgcIl-12 isoforms on Th17-related cytokines mRNA expression in grass carp lymphocytes. Lymphocytes were treated with 30-1000 ng/mL rgcIl-12AB **(A, B)** or rgcIl-12BB **(C, D)** for 12 h. The mRNA expression of *il-17a/f1* and *il-22* mRNA were detected by qPCR and normalized by *β-actin* and expressed as fold changes compared with the control group. Data are shown as mean ± SEM (N = 4). *p < 0.05, **p < 0.01, and ***p < 0.001.

In lymphocytes, both rgcIl-12AB and rgcIl-12BB were effective in enhancing the Th17 related cytokines *il-17a/f1* and *il-22* mRNA expression from 30 to 1000 ng/mL (**Figures 7A–D**). At the same time, in monocytes/macrophages, the different concentrations (30-1000 ng/mL) of rgcIl-12AB and rgcIl-12BB had no effect on the expression of *il-17a/f1* and *il-22* (**Figures S12A–D**).

### Signaling Transduction Mechanisms for gcIl-12 Isoforms-Stimulated Il-17a/f1 Transcription in Grass Carp Lymphocytes

**Figure 8:**
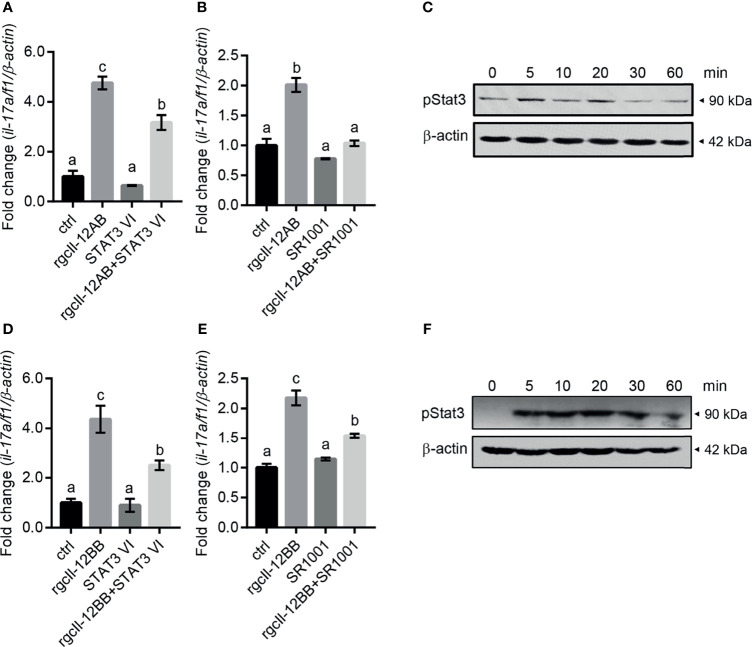
Signaling pathways of rgcIl-12 isoforms in regulating *il-17a/f1* in lymphocytes. **(A)** Lymphocytes were incubated with rgcIl-12AB (1000 ng/mL) in the presence or absence of STAT3 VI (STAT3 inhibitor, 30 µM). **(B)** Lymphocytes were incubated with RgcIl-12AB (1000 ng/mL) in the presence or absence of SR1001 (RORγt inhibitor, 15 µM). The *il-17a/f1* mRNA was normalized by *β-actin* and expressed as fold changes compared with the control group. **(C)** Phosphorylation of Stat3 was detected in the lymphocytes treated with rgcIl-12AB (1000 ng/mL) for 0-60 min. **(D)** Lymphocytes were incubated with rgcIl-12BB (1000 ng/mL) in the presence or absence of STAT3 VI (STAT3 inhibitor, 30 µM). **(E)** Lymphocytes were incubated with rgcIl-12BB (1000 ng/mL) in the presence or absence of SR1001 (RORγt inhibitor, 15 µM). The *il-17a/f1* mRNA was normalized by *β-actin* and expressed as fold changes compared with the control group. Data are shown as mean ± SEM (N = 4). Different letters indicate significant differences at *p* < 0.05. **(F)** Phosphorylation of Stat3 was detected in lymphocytes treated with rgcIl-12BB (1000 ng/mL) for 0-60 min.

To elucidate the signaling mechanisms in the modulation of rgcIl-12 isoforms on *il-17a/f1* transcription, the inhibitors for Stat3 and Rorγt signaling were used in the present study. Results showed that the stimulatory effects of rgcIl-12AB and rgcIl-12BB (1000 ng/mL) on the *il-17a/f1* mRNA expression were impeded by STAT3 VI (30 μM, STAT3 inhibitor) (**Figures 8A, D**) and SR1001 (15 μM, RORγt inhibitor) (**Figures 8B, E**). In addition, rgcIl-12AB (1000 ng/mL) was able to induce the phosphorylation of Stat3 from 5 to 20 min (**Figure 8C**) and rgcIl-12BB (1000 ng/mL) was able to induce the phosphorylation of Stat3 from 5 to 60 min (**Figure 8F**).

### Modification of gcTgf-β1 on the Regulatory Effects of gcIl-12 Isoforms in Grass Carp Lymphocytes

**Figure 9:**
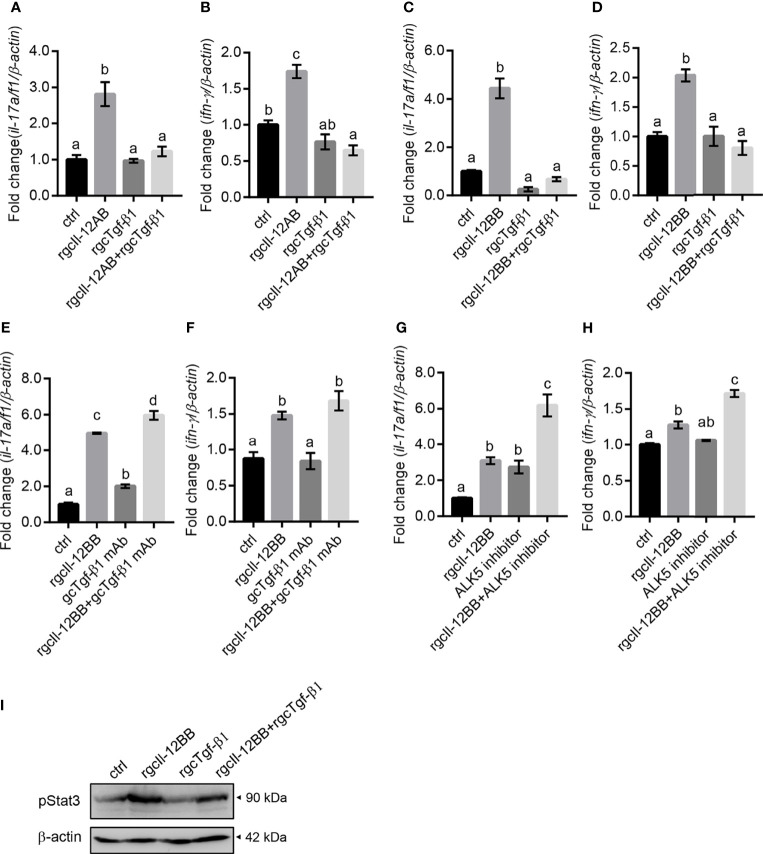
The effects of gcTgf-β1 on rgcIl-12AB- and rgcIl-12BB-induced *il-17a/f1* and *ifn-γ* mRNA expression. Lymphocytes were treated with 1000 ng/mL of rgcIl-12AB **(A, B)** and rgcIl-12BB **(C, D)** in the presence or absence of rgcTgf-β1 (100 ng/mL) for 12 h. **(E, F)** Lymphocytes were exposed to rgcIl-12BB (1000 ng/mL) in the presence or absence of gcTgf-β1 mAb (1:1000 diluted) for 12 h. **(G, H)** Lymphocytes were incubated with rgcIl-12BB (1000 ng/mL) in the presence or absence of ALK5 inhibitor (TGF-β1 RI Kinase inhibitor VIII, 2 µM) for 12 h. Then the levels of *il-17a/f1* and *ifn-γ* mRNA were detected by qPCR. Relative mRNA levels were analyzed using *β-actin* as an internal reference and expressed as the fold changes of the control group (blank column). Data are shown as mean ± SEM (N = 4). Different letters indicate significant differences at *p* < 0.05. **(I)** Phosphorylation of Stat3 was detected in lymphocytes incubated with rgcIl-12BB (1000 ng/mL) in the presence or absence of rgcTgf-β1 (100 ng/mL) for 20 min.

To assess whether the regulatory effects of gcIl-12 isoforms were modified in lymphocytes, the involvement of gcTgf-β1 in mediating these effects was examined. RgcTgf-β1 (100 ng/mL) significantly inhibited rgcIl-12AB-stimulated *il-17a/f1* and *ifn-γ* gene expression (**Figures 9A, B**) and rgcTgf-β1 also significantly inhibited rgcIl-12BB-elevated *il-17a/f1* and *ifn-γ* gene expression (**Figures 9C, D**). Immunoneutralization of gcTgf-β1 secreted from the cells by using an anti-gcTgf-β1 mAb (1:2000 diluted) could enhance *il-17a/f1* mRNA expression alone or in combination with rgcIl-12BB (**Figure 9E**), but immunoneutralization of gcTgf-β1 had no effect on *ifn-γ* mRNA expression (**Figure 9F**). Moreover, ALK5 inhibitor (2 µM) was able to up-regulate *il-17a/f1* but not *ifn-γ* mRNA expression alone, and further stimulated rgcIl-12BB-induced *il-17a/f1* and *ifn-γ* transcription (**Figures 9G, H**). Additionally, rgcTgf-β1 (100 ng/mL) was effective in reducing rgcIl-12BB-triggered phosphorylation of Stat3 in the cells (**Figure 9I**).

## Discussion

Teleost-specific WGD generates two or three *p35* paralogues in different fish species (10). In addition to one *gcp35* gene reported previously (15), we found another *p35* gene in grass carp (**Figure S1**), and these *gcp35* paralogues (**Figure 1**) may have arisen from a common ancestral gene. These findings raised a question of whether the roles of distinct *p35* paralogues are the same. Given that p35 subunit is a rate-limiting factor for the heterodimeric IL-12 generation in both fish and mammals (4, 11, 14, 34, 35), the inducible expression patterns of *p35* may determine the role of *p35* paralogues (11). In rainbow trout and amberjack, bacteria are able to stimulate both *p35a* and *p35b* transcription in spleen and head kidney leukocytes (12, 13), indicating a possible involvement of *p35* paralogues in the inflammatory response to pathogen challenges. However, these findings do not provide information on the discrepancy of their roles in fish. Up to now, modulation on the expression of three *p35* paralogues (*p35a1*, *p35a2* and *p35b*) by LPS, poly I:C, and pro-inflammatory cytokines has been observed in head-kidney cells of Atlantic salmon (11). However, these stimulatory effects on *p35* paralogues expression are minor except that poly I:C, which markedly induced the expression of *p35a1* and *p35a2*. This was potentially due to the mixed cell types in head-kidney cells as described by the authors (11). In the present study, we used grass carp monocytes/macrophages as a cell model considering that macrophages are the major physiological producers of Il-12 (36, 37). Results showed that poly I:C and rgcIfn-γ visibly augmented the expression of *gcp35a* and *gcp35b*, respectively, and we elucidated the signaling mechanisms responsive for these findings (**Figure 2**). It is noteworthy that the dramatic up-regulation of poly I:C on *p35a* but not *p35b* paralogue is observed in both grass carp and Atlantic salmon (11), suggesting the role of *p35a* paralogue in response to viral infection in fish. In view of IFN-γ being the major target of IL-12, the intense stimulation of rgcIfn-γ on *gcp35b* expression indicated a “positive feedback loop” by local interaction between Il-12 and Ifn-γ in fish, thereby reinforcing the role of *p35b* in magnifying or maintaining Il-12 and Ifn-γ signaling. In addition, LPS had no effect on both *gcp35* genes, but it can significantly up-regulate the mRNA levels of *gcp40* paralogues in periphery blood lymphocytes (16) and their protein secretion in HKLs (30), indicating different roles of p35 and p40 subunits in fish. In fact, our previous studies have reported that gcp40 subunits can be independently released and have their own functions (23, 30) as seen in mammals (38, 39). Our data provide a clue for understanding the diverse roles of Il-12 isoforms in fish immunity.

In mammals, it is well known that IL-12 is a disulfide-bridged heterodimer comprising a p35 and a p40 subunit (1, 40). Using grouper as the model, Tsai et al. suggest that the inter-chain disulfide bond between IL-12 subunits is conserved from teleosts to mammals IL-12 (41). In agreement with this, our results together with other reports disclosed the existence of the conserved cysteine residues in p35 and p40 subunits which can potentially form the inter-chain disulfide bond in grass carp and other fish species (**Figure S2**) (16, 41–43). These findings prompted us to perform 3D structural modeling analysis, and the relative reliability of the predicted models was supported by QMEANDisCo Global scores. Results showed that gcp40c could not form heterodimers due to lacking the cysteine residues responsible for the formation of inter-chain disulfide bond. Similarly, common carp p40c also lacks the key cysteine residue to form the inter-chain disulfide bond with common carp p35 subunit (43). Accordingly, it is possible that there are four gcIl-12 heterodimers (gcp35a/gcp40a, gcp35a/gcp40b, gcp35b/gcp40a and gcp35b/gcp40b). Furthermore, given that the secreted Il-12 reflects the existence of heterodimer, we detected the protein samples from the culture media of HEK293 cells transfected with gcp35a/b-FLAG and gcp40a/b/c-HIS and analyzed under non-reducing conditions, thereby providing direct evidence for the generation of gcIl-12 isoforms. As shown in **Figure 4**, the MWs of gcp35a (35 kDa), gcp35b (25 kDa), gcp40a (55 kDa), gcp40b (50 kDa) and gcp40c (43 kDa) were bigger than their predicted sizes (gcp35a, 23 kDa; gcp35b, 23 kDa; gcp40a, 40 kDa; gcp40b, 39 kDa and gcp40c, 37 kDa) with varying degrees, indicating the occurrence of glycosylation of these subunits. In fact, IL-12 is generally considered as a glycoprotein (14, 40, 44), and it has been demonstrated that the glycosylation of IL-12 family cytokines affects the biogenesis and function of these cytokines (45). The gcp40c did not form heterodimers with two gcp35 paralogues, confirming the notion that gcp40c cannot form an inter-chain disulfide bond. In addition, it is worth mentioning that a band with more than 100 kDa of MW was detected by anti-HIS antibody (**Figure 4B**) but not anti-FLAG antibody (**Figure 4A**) in the lanes loaded with the sample of gcp40a and gcp35a/b overexpression, indicating the preferential formation of gcp40a homodimer. This might result in an obstacle to generate heterodimer of gcp40a with gcp35a/b. Taken together, it was suggested that only two combinations of the subunits (gcp35a/gcp40b and gcp35b/gcp40b) could form Il-12 heterodimers. In the past years, although the recombinant fish Il-12 isoforms have been prepared based on the co-expression of p35 and p40 subunits (12, 14), the evidence for the existence of heterodimeric Il-12 isoforms is still lacking. Our results uncovered that the generation of Il-12 isoforms is selective although there are multiple *p35* and *p40* paralogues in fish. This figures out the direction to prepare recombinant Il-12 proteins.

Along this line, rgcIl-12AB and rgcIl-12BB were prepared in CHO cells and their sizes were bigger than the predicted MWs (**Figure S7**). This is in agreement with the results of the glycosylation site prediction of gcp35a/b and gcp40b (**Figure S3**) and the glycosidase digestion assay (**Figure S8**). Consistent with the classical function of IL-12 to regulate IFN-γ expression (46, 47), rgcIl-12AB and rgcIl-12BB could increase *ifn-γ* gene expression and secretion in grass carp HKLs (**Figure 5**). This finding was further confirmed by the potential of gcIl-12AB and gcIl-12BB to stimulate *ifn-γ* transcription in both lymphocytes and monocytes/macrophages (**Figure 6**), strengthening the role of gcIl-12 isoforms in host defense *via* inducing Ifn-γ production as seen in mammals (48–50).

Unexpectedly, rgcIl-12AB and rgcIl-12BB were effective in modulating the *il-17a/f1* gene expression in lymphocytes but not monocytes/macrophages, indicating the novel function of Il-12 involving in Th17-like response in fish for the first time. In mammals, it is well known that as a hallmark cytokine of the Th17 cell, IL-17 production is mainly manipulated by IL-23 through STAT3/RORγt signaling pathways (51, 52). In teleosts, our previous study has proved that grass carp Il-23 isoforms also display the ability to trigger Th17-like response by Stat3 signaling (53). In this study, our results suggested that rgcIl-12 isoforms possessed a similar function to Il-23, and this notion was supported by the analysis of the signaling mechanisms that rgcIl-12BB up-regulated *il-17a/f1* transcription through Stat3/Rorγt pathways in lymphocytes (**Figure 8**). Intriguingly, grass carp p40 isoforms also show the potential to mediate Th17-like responses by similar signaling pathways (23). Accordingly, the rgcIl-12 isoforms shared the same regulatory function with rgcIl-23 and rgcp40 isoforms on Th17-like responses. These findings raised a possibility that common Il-12/23p40 receptor (Il-12rβ1) signaling may play a role in mediating Th17-like response and indicated the important role of Il-17a/f1 in fish immunity. In agreement with this notion, mammalian IL-17 can effectively recruit neutrophils (54) and regulate tissue inflammation such as intestinal inflammation (55), and our previous study suggests that gcIl-17a/f1 can recruit immune cells through producing chemokine Cxcl-8 (56). Additionally, the pleiotropic properties of gcIl-12AB and gcIl-12BB prompted us to find a way to control gcIl-12 signaling. Fortunately, in accordance with the role of TGF-β1 as a classical negative regulator for IL-12 signal in IFN-γ production in mammals (57), we found that rgcTgf-β1 suppressed two gcIl-12 isoforms-stimulated transcription of *il-17a/f1* and *ifn-γ via* Stat3 signaling in grass carp lymphocytes (**Figure 9**), suggesting an intrinsic regulatory route for restricting Il-12 signaling in fish.

Taken together, our works uncovered different expression patterns of two *gcp35* paralogues and the exact composition of gcIl-12 isoforms, and explored a new function of gcIl-12 isoforms. These data provide new insights into the generation and function of heterodimeric cytokine isoforms expanded by teleost-specific WGD events during evolution.

## Data Availability Statement

The datasets presented in this study can be found in online repositories. The names of the repository/repositories and accession number(s) can be found in the article/**Supplementary Material**.

## Ethics Statement

The animal study was reviewed and approved by University of Electronic Science and Technology of China Experimentation Ethics Review Committee.

## Author Contributions

HZ and XQ conceived and designed the experiments. XQ performed the experiments and analyzed the data. HZ and XQ wrote the manuscript. XW, AZ, and KY participated in the design of the study. HS, DW, and JR helped analyzed experiments and data. HZ edited the manuscript and provided reagents and experiment space. All authors contributed to the article and approved the submitted version.

## Funding

This work was supported by the grant from the National Natural Science Foundation of China (31572650).

## Conflict of Interest

The authors declare that the research was conducted in the absence of any commercial or financial relationships that could be construed as a potential conflict of interest.

## Publisher’s Note

All claims expressed in this article are solely those of the authors and do not necessarily represent those of their affiliated organizations, or those of the publisher, the editors and the reviewers. Any product that may be evaluated in this article, or claim that may be made by its manufacturer, is not guaranteed or endorsed by the publisher.

## Supplementary Material

Supplementary Figure 1 Nucleotide and amino acid sequence of *gcp35b*. The nucleotides in bold indicate the start codon (ATG) and the stop codon (TGA). The putative amino acid sequence is shown under the triplet codon. The predicted signal peptide is underlined.

Supplementary Figure 2 **(A)** Alignment of p35 amino acid sequences of grass carp, zebrafish, fugu, frog, chicken, mouse and human. Conserved cysteines involved in the formation of intra-chain or inter-chain disulfide bonds are shaded by yellow. The cysteine residue that forms an inter-chain disulfide bridge with p40 is indicated by the star. The gcp35a conserved cysteine residue sites are marked with red letters, while the blue letters indicate the gcp35b conserved cysteine sites. **(B)** Alignment of p40 amino acid sequences of grass carp, chicken, mouse and human. Conserved cysteine residues are shown in yellow background and that marked with the same number are involved in the formation of intra-chain disulfide bonds. The conserved cysteine residue of gcp40 that form an inter-chain disulfide bond with gcp35 is denoted with star. And the inter-chain disulfide bond cysteine sites of gcp40a, gcp40b and gcp40c are marked with red, blue and black letters, separately.

Supplementary Figure 3 The glycosylation sites analysis of grass carp Il-12 subunits. Asn-Xaa-Ser/Thr sequons in the sequence output below are highlighted in blue. Asparagines predicted to be N-glycosylated are highlighted in red.

Supplementary Figure 4 Inductive expression of *gcp35a* and *gcp35b* in grass carp monocytes/macrophages treated by poly I:C and rgcIfn-γ for different times. The monocytes/macrophages were treated with poly I:C (50 µg/mL) **(A)** and rgcIfn-γ (500 ng/mL) **(B)** for 1, 6 and 12 h. The “n.s.” indicates no significant difference, ***p* < 0.01, and ****p* < 0.001. Data are shown as mean ± SEM (N = 4).

Supplementary Figure 5 Molecular weight prediction and amino acid sequence alignments of the signaling molecules. **(A)** The predicted sizes of grass carp, human and zebrafish signaling molecules. **(B)** The amino acid residues surrounding phosphorylation sites of grass carp signaling molecules were aligned with the human or zebrafish homologues.

Supplementary Figure 6 The specificity of anti-gcp35a and gcp35b pAb was validated by Western blotting. **(A)** Western blotting analysis by using anti-gcp35a pAb (1:1000 diluted). **(B)** Western blotting analysis by using anti-gcp35a pAb (1:1000 diluted) pre-absorbed with 3 μg of rgcp35a. **(C)** Western blotting analysis by using anti-gcp35b pAb (1:1000 diluted). **(D)** Western blotting analysis by using anti-gcp35a pAb (1:1000 diluted) pre-absorbed with 3 μg of rgcp35b. (Lane 1, 3: The rgcp35a; lane 2, 4, 5 and 7: The cell lysis of grass carp HKLs; lane 6, 8: The cell lysis of grass carp HKLs treated with HI *A. hydrophila*, MOI 1:1).

Supplementary Figure 7 SDS-PAGE and WB analysis of rgcIl-12AB and rgcIl-12BB. **(A)** SDS-PAGE analysis of rgcIl-12AB. **(B, C)** Verification of the rgcIl-12AB by western blotting analysis using anti-gcp35a pAb (1:1000) and anti-gcp40b pAb (1:1000). **(D)** SDS-PAGE analysis of rgcIl-12BB. **(E, F)** Verification of the rgcIl-12BB by western blotting analysis using anti-gcp35b pAb (1:1000) and gcp40b pAb (1:1000). Marker: molecular weight marker; lane 1: total proteins before purification; lane 2: the flow-through sample; lane 3: the proteins washed by 50 mM imidazole; lane 4: the proteins washed by 500 mM imidazole and lane 5: the purified protein.

Supplementary Figure 8 Verification of rgcIl-12 isoforms’ glycosylation. **(A)** SDS-PAGE analysis of rgcIl-12AB glycosylation. **(B)** SDS-PAGE analysis of rgcIl-12BB glycosylation. **(C)** WB analysis of rgcIl-12AB digested by glycosidase. **(D)** WB analysis of rgcIl-12BB digested by glycosidase. The MW of glycosidase (PNGase F) is 36 kDa.

Supplementary Figure 9 Effects of rgcIl-12BB on *ifn-γ* mRNA expression in grass carp HKLs. HKLs were treated with 1000 ng/mL of rgcIl-12BB for 1, 6, 12 and 24 h, separately. The mRNA expression of *ifn-γ* was detected by RT-qPCR, and the relative mRNA levels were normalized by *β-actin* and expressed as fold changes compared with the control group of each time point. ***p* < 0.01, and ****p* < 0.001. Data are shown as mean ± SEM (N = 4).

Supplementary Figure 10 The specificity of anti-gcIfn-γ pAb was validated by Western blotting. **(A)** SDS-PAGE analysis of rgcIfn-γ. **(B)** Western blotting analysis by using anti-gcIfn-γ pAb (1:1000 diluted). **(C)** Western blotting analysis by using anti-gcIfn-γ pAb (1:1000 diluted) pre-absorbed with 3 μg of rgcIfn-γ.

Supplementary Figure 11 The gcIfn-γ competitive-inhibition ELISA standard curve. **(A)** The original data of gcIfn-γ competitive-inhibition ELISA standard. The inhibition ratio was calculated by the formula: Inhibition ratio = 100% × [OD450 (0 ng/mL) - OD450 (other concentration)]/OD450 (0 ng/mL). **(B)** The standard curve was drawn according to the inhibition ratio and the corresponding protein concentrations.

Supplementary Figure 12 Effects of rgcIl-12 isoforms on *il-17a/f1* and *il-22* mRNA expression in grass carp monocytes/macrophages. Grass carp monocytes/macrophages were treated with 30-1000 ng/mL rgcIl-12AB **(A, B)** or rgcIl-12BB **(C, D)** for 12 h. The mRNA expression of *il-17a/f1* and *il-22* was detected by RT-qPCR and the relative mRNA levels were normalized by *β-actin* and expressed as fold changes compared with the control group. Data are shown as mean ± SEM (N=4). The “n.s.” indicates no significant.

Supplementary Table 1 The primers used in the present study.

Supplementary Table 2 QMEANDisCo Global score of the predicted models.
