# Supplementary figures and images for: Stimulus-Specific Expression, Selective Generation and Novel Function of Grass Carp (Ctenopharyngodon idella) IL-12 Isoforms: New Insights Into the Heterodimeric Cytokines in Teleosts
Source: Front Immunol. 2021 Sep 16;12:734535. doi: 10.3389/fimmu.2021.734535 (PMC8481787; doi:10.3389/fimmu.2021.734535)

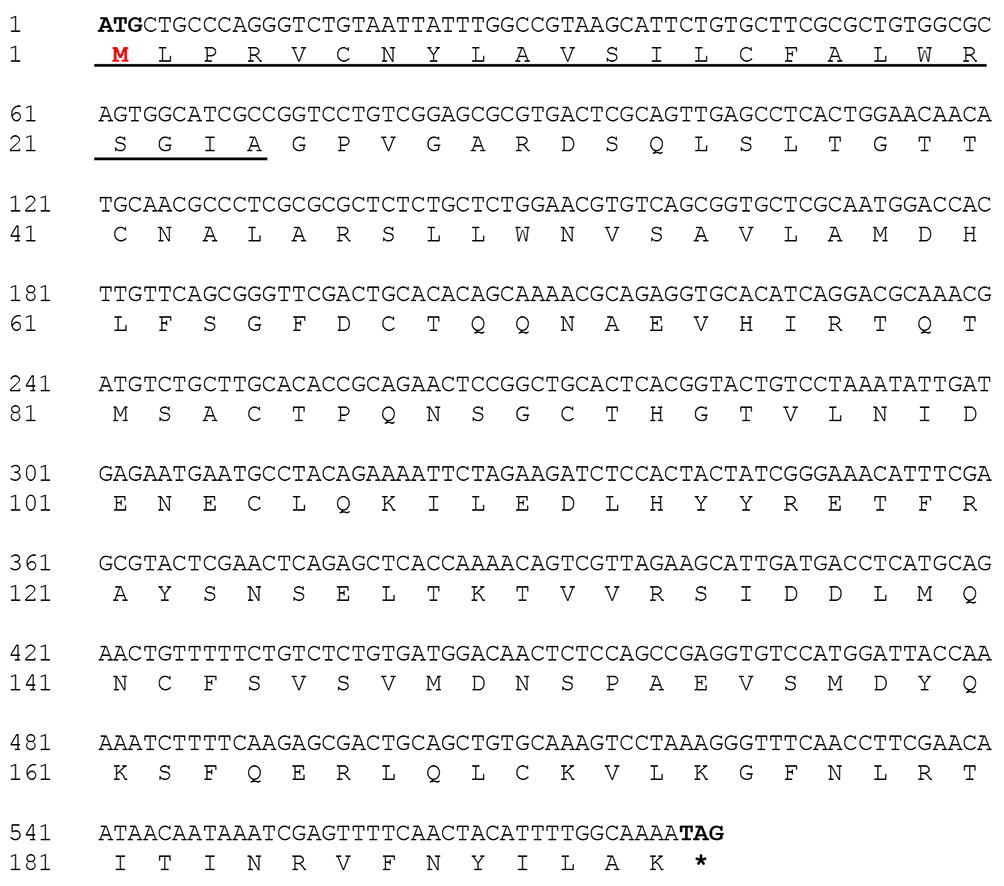

Supplement: Supplementary Figure 1 — Nucleotide and amino acid sequence of gcp35b. The nucleotides in bold indicate the start codon (ATG) and the stop codon (TGA). The putative amino acid sequence is shown under the triplet codon. The predicted signal peptide is underlined. [file Image_1.tif]

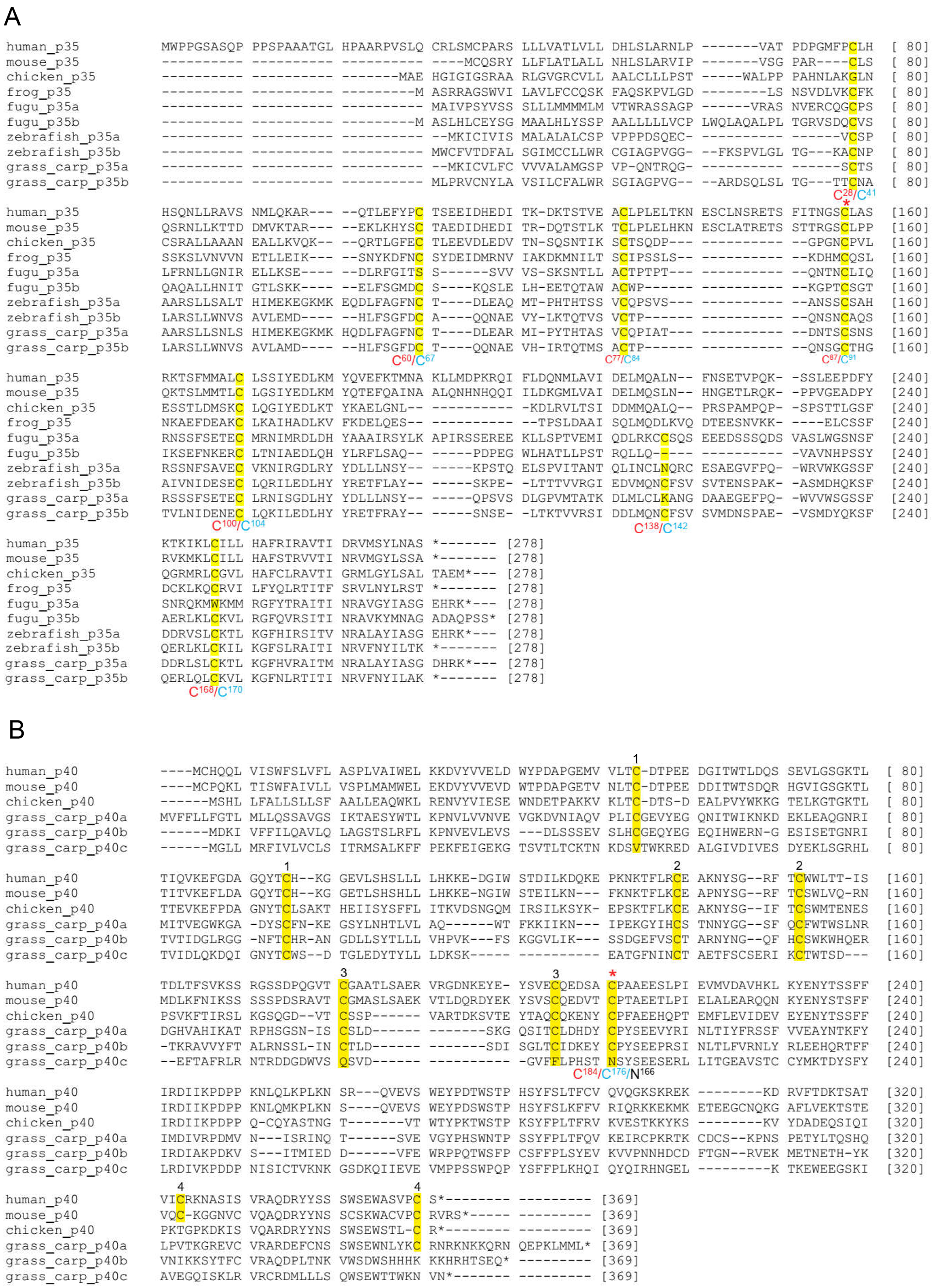

Supplement: Supplementary Figure 2 — (A) Alignment of p35 amino acid sequences of grass carp, zebrafish, fugu, frog, chicken, mouse and human. Conserved cysteines involved in the formation of intra-chain or inter-chain disulfide bonds are shaded by yellow. The cysteine residue that forms an inter-chain disulfide bridge with p40 is indicated by the star. The gcp35a conserved cysteine residue sites are marked with red letters, while the blue letters indicate the gcp35b conserved cysteine sites. (B) Alignment of p40 amino acid sequences of grass carp, chicken, mouse and human. Conserved cysteine residues are shown in yellow background and that marked with the same number are involved in the formation of intra-chain disulfide bonds. The conserved cysteine residue of gcp40 that form an inter-chain disulfide bond with gcp35 is denoted with star. And the inter-chain disulfide bond cysteine sites of gcp40a, gcp40b and gcp40c are marked with red, blue and black letters, separately. [file Image_2.tif]

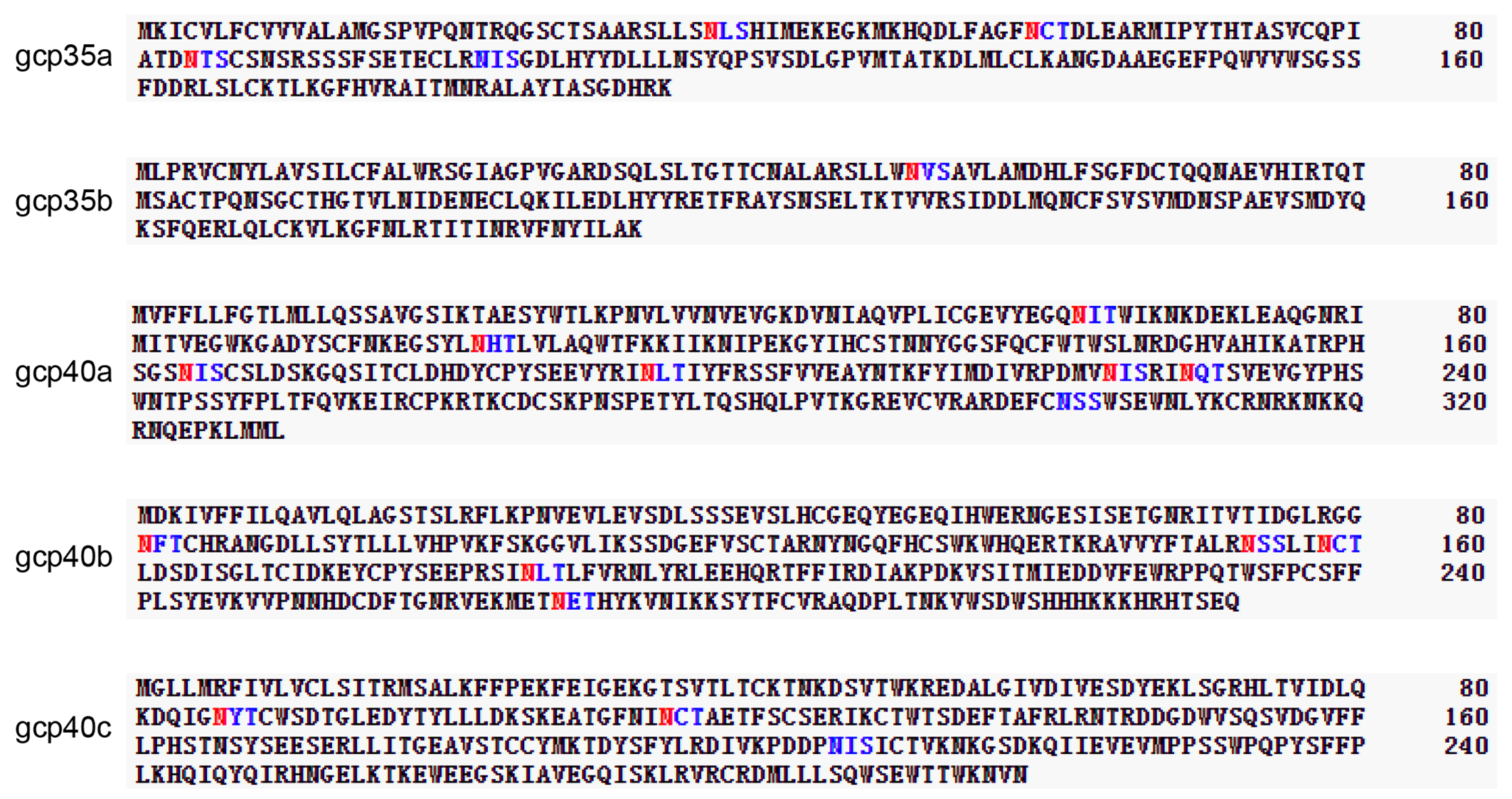

Supplement: Supplementary Figure 3 — The glycosylation sites analysis of grass carp Il-12 subunits. Asn-Xaa-Ser/Thr sequons in the sequence output below are highlighted in blue. Asparagines predicted to be N-glycosylated are highlighted in red. [file Image_3.tif]

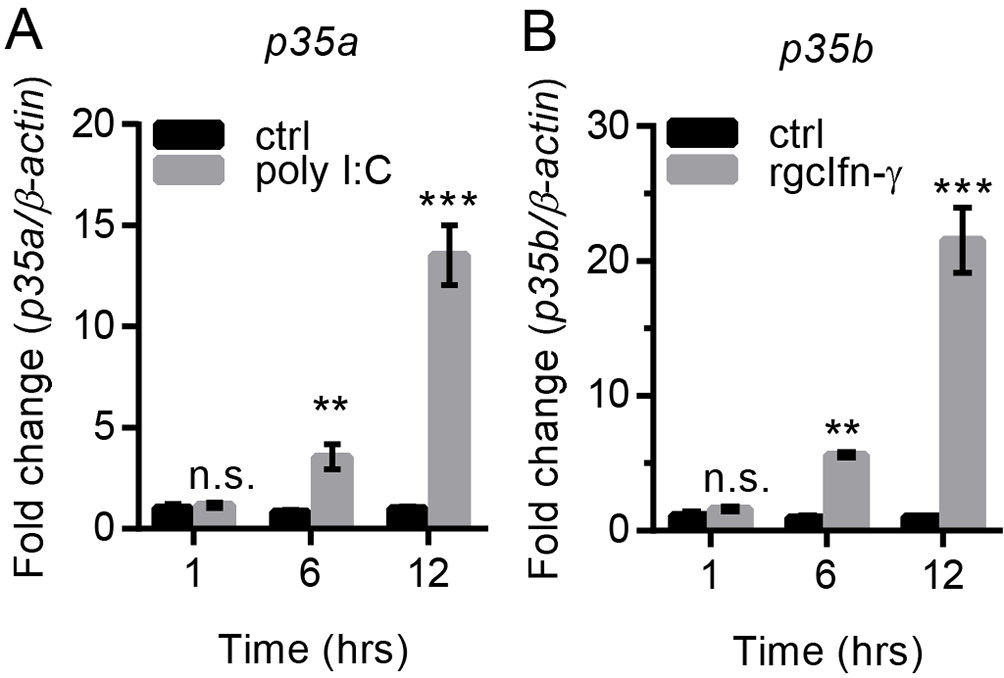

Supplement: Supplementary Figure 4 — Inductive expression of gcp35a and gcp35b in grass carp monocytes/macrophages treated by poly I:C and rgcIfn-γ for different times. The monocytes/macrophages were treated with poly I:C (50 µg/mL) (A) and rgcIfn-γ (500 ng/mL) (B) for 1, 6 and 12 h. The “n.s.” indicates no significant difference, **p < 0.01, and ***p < 0.001. Data are shown as mean ± SEM (N = 4). [file Image_4.tif]

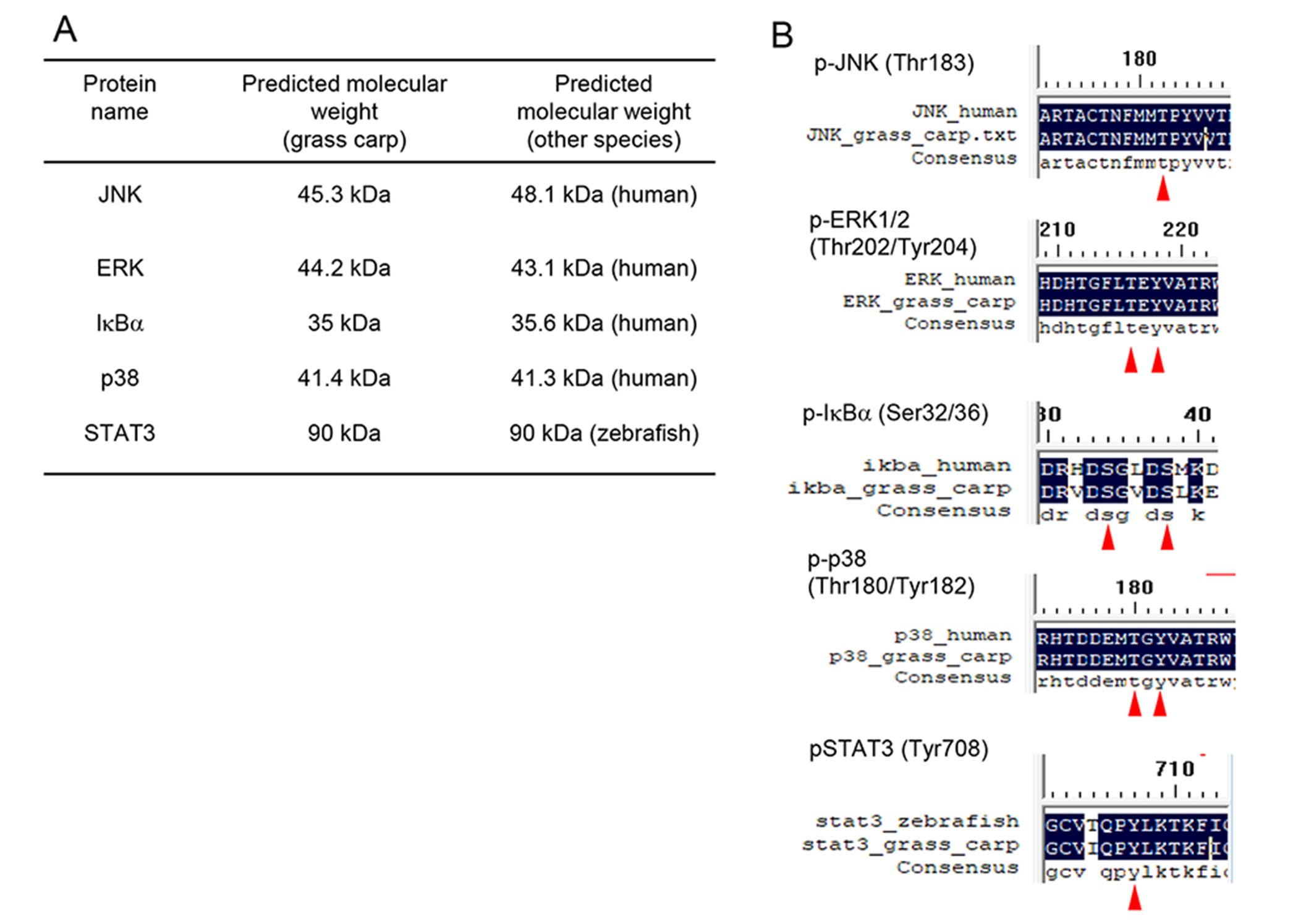

Supplement: Supplementary Figure 5 — Molecular weight prediction and amino acid sequence alignments of the signaling molecules. (A) The predicted sizes of grass carp, human and zebrafish signaling molecules. (B) The amino acid residues surrounding phosphorylation sites of grass carp signaling molecules were aligned with the human or zebrafish homologues. [file Image_5.tif]

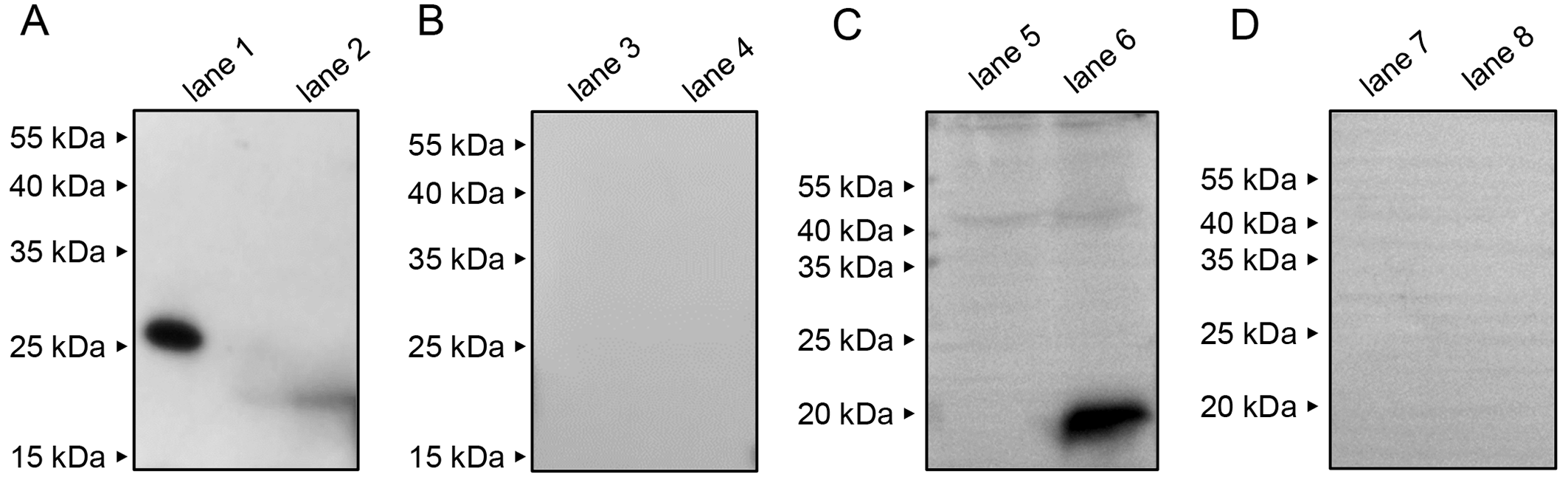

Supplement: Supplementary Figure 6 — The specificity of anti-gcp35a and gcp35b pAb was validated by Western blotting. (A) Western blotting analysis by using anti-gcp35a pAb (1:1000 diluted). (B) Western blotting analysis by using anti-gcp35a pAb (1:1000 diluted) pre-absorbed with 3 μg of rgcp35a. (C) Western blotting analysis by using anti-gcp35b pAb (1:1000 diluted). (D) Western blotting analysis by using anti-gcp35a pAb (1:1000 diluted) pre-absorbed with 3 μg of rgcp35b. (Lane 1, 3: The rgcp35a; lane 2, 4, 5 and 7: The cell lysis of grass carp HKLs; lane 6, 8: The cell lysis of grass carp HKLs treated with HI A. hydrophila, MOI 1:1). [file Image_6.tif]

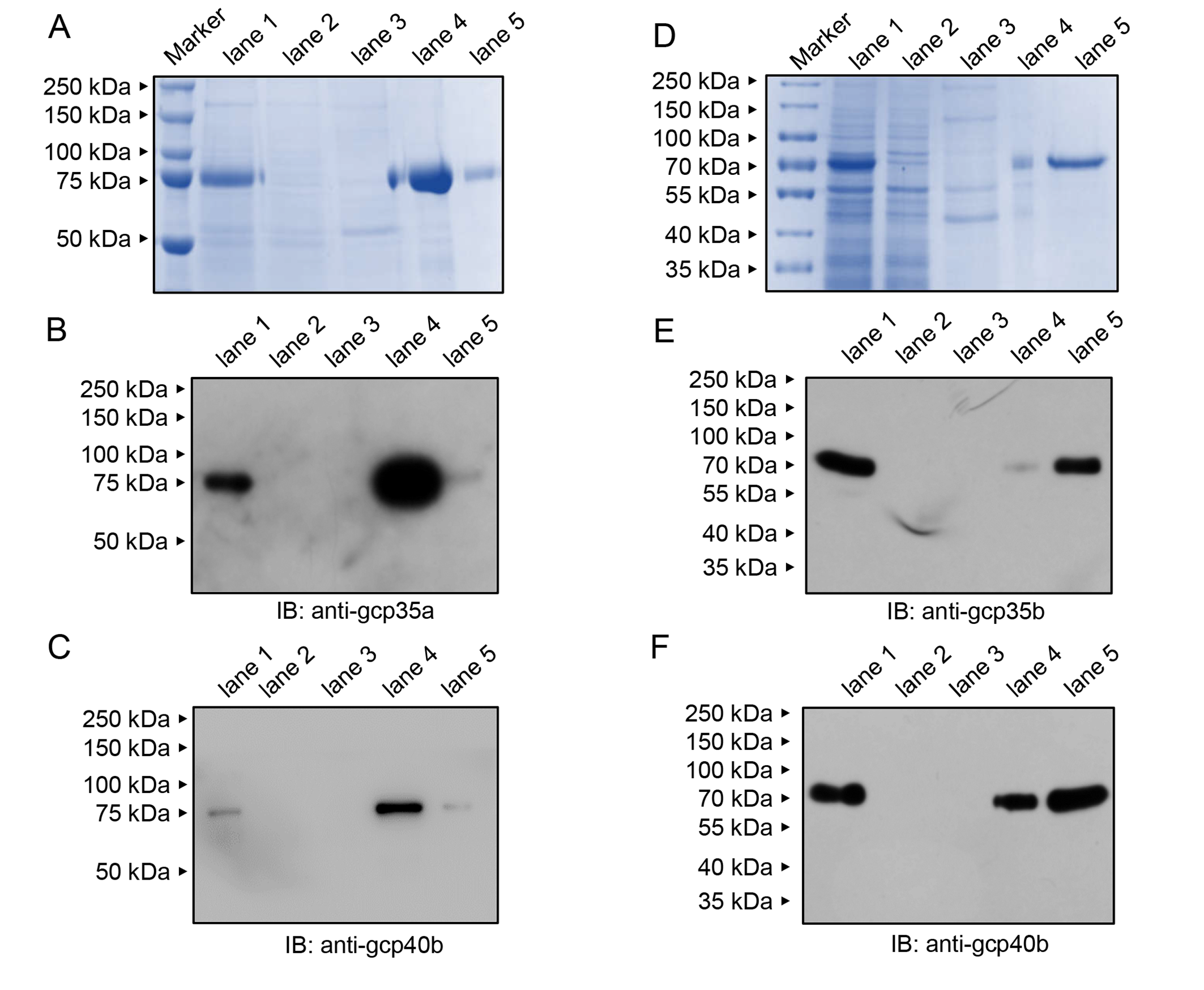

Supplement: Supplementary Figure 7 — SDS-PAGE and WB analysis of rgcIl-12AB and rgcIl-12BB. (A) SDS-PAGE analysis of rgcIl-12AB. (B, C) Verification of the rgcIl-12AB by western blotting analysis using anti-gcp35a pAb (1:1000) and anti-gcp40b pAb (1:1000). (D) SDS-PAGE analysis of rgcIl-12BB. (E, F) Verification of the rgcIl-12BB by western blotting analysis using anti-gcp35b pAb (1:1000) and gcp40b pAb (1:1000). Marker: molecular weight marker; lane 1: total proteins before purification; lane 2: the flow-through sample; lane 3: the proteins washed by 50 mM imidazole; lane 4: the proteins washed by 500 mM imidazole and lane 5: the purified protein. [file Image_7.tif]

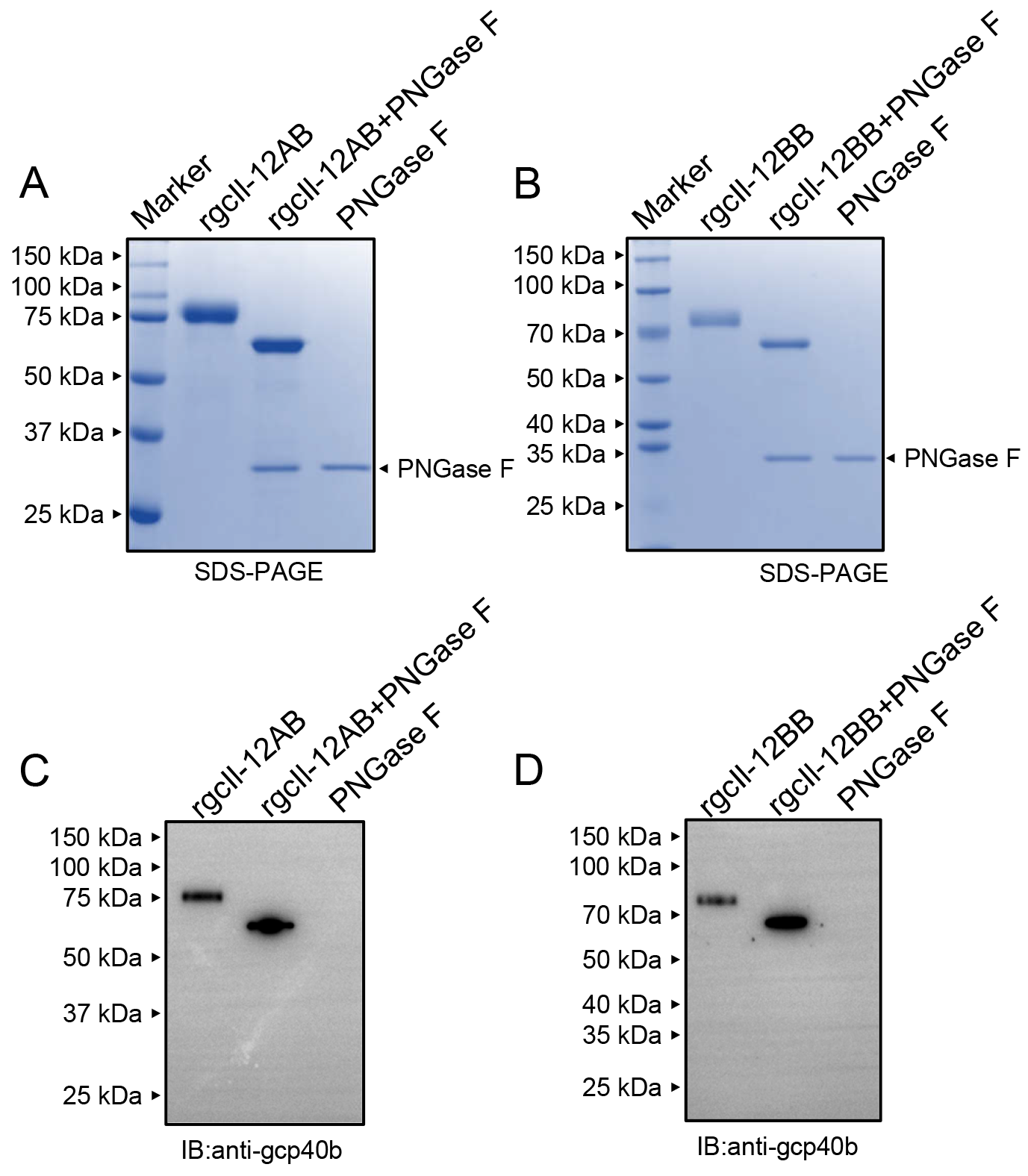

Supplement: Supplementary Figure 8 — Verification of rgcIl-12 isoforms’ glycosylation. (A) SDS-PAGE analysis of rgcIl-12AB glycosylation. (B) SDS-PAGE analysis of rgcIl-12BB glycosylation. (C) WB analysis of rgcIl-12AB digested by glycosidase. (D) WB analysis of rgcIl-12BB digested by glycosidase. The MW of glycosidase (PNGase F) is 36 kDa. [file Image_8.tif]

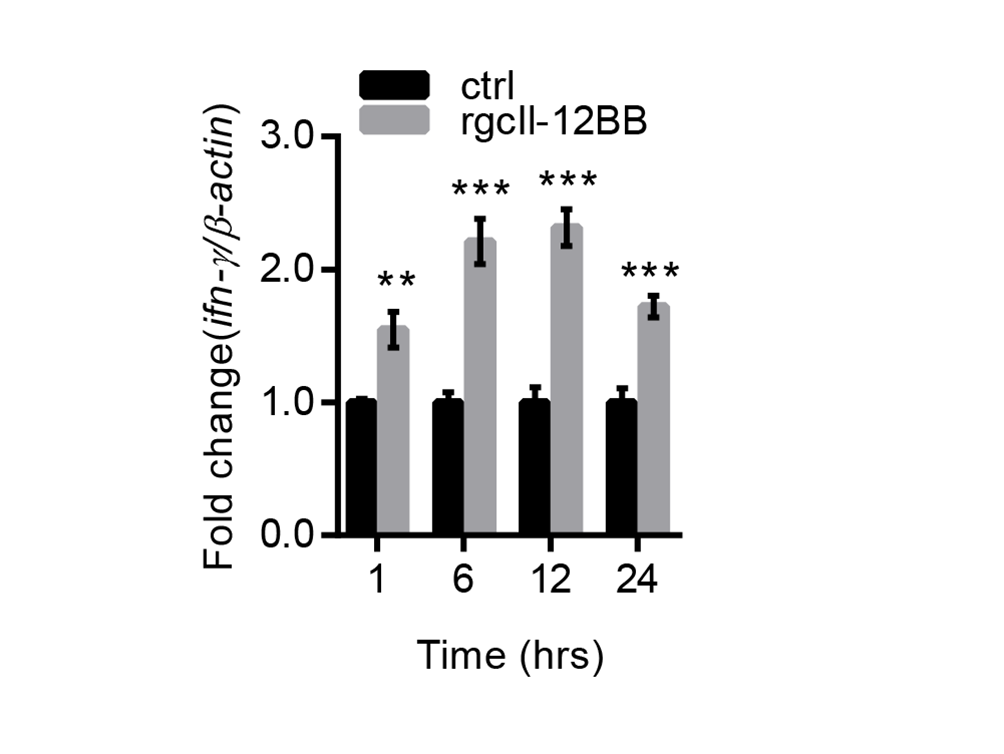

Supplement: Supplementary Figure 9 — Effects of rgcIl-12BB on ifn-γ mRNA expression in grass carp HKLs. HKLs were treated with 1000 ng/mL of rgcIl-12BB for 1, 6, 12 and 24 h, separately. The mRNA expression of ifn-γ was detected by RT-qPCR, and the relative mRNA levels were normalized by β-actin and expressed as fold changes compared with the control group of each time point. **p < 0.01, and ***p < 0.001. Data are shown as mean ± SEM (N = 4). [file Image_9.tif]

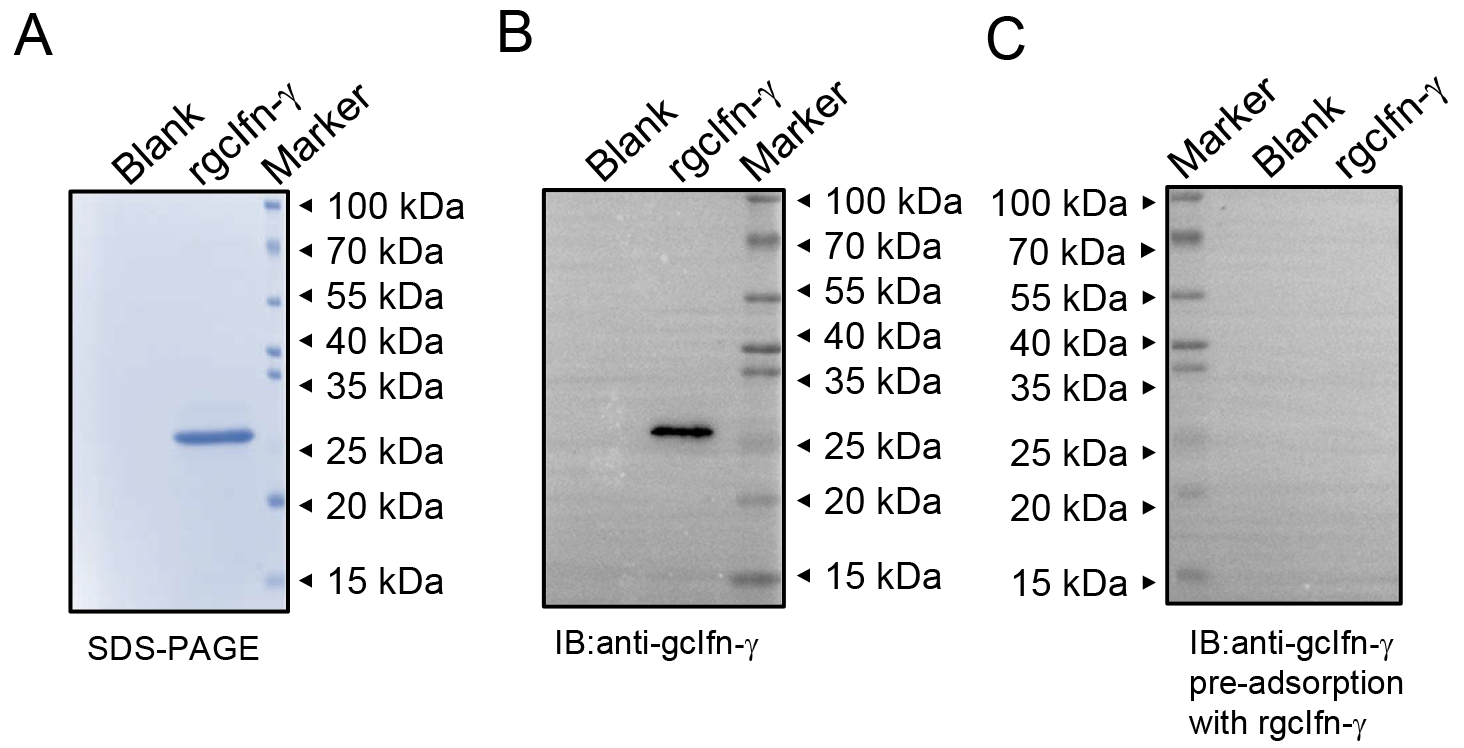

Supplement: Supplementary Figure 10 — The specificity of anti-gcIfn-γ pAb was validated by Western blotting. (A) SDS-PAGE analysis of rgcIfn-γ. (B) Western blotting analysis by using anti-gcIfn-γ pAb (1:1000 diluted). (C) Western blotting analysis by using anti-gcIfn-γ pAb (1:1000 diluted) pre-absorbed with 3 μg of rgcIfn-γ. [file Image_10.tif]

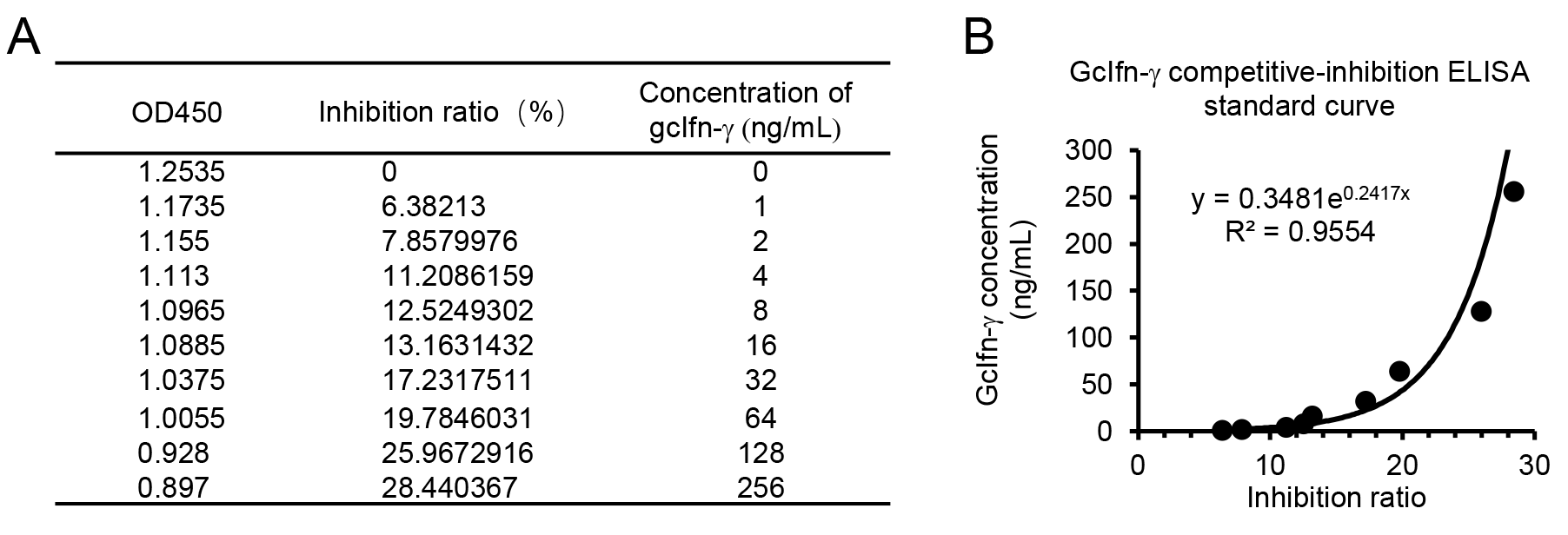

Supplement: Supplementary Figure 11 — The gcIfn-γ competitive-inhibition ELISA standard curve. (A) The original data of gcIfn-γ competitive-inhibition ELISA standard. The inhibition ratio was calculated by the formula: Inhibition ratio = 100% × [OD450 (0 ng/mL) - OD450 (other concentration)]/OD450 (0 ng/mL). (B) The standard curve was drawn according to the inhibition ratio and the corresponding protein concentrations. [file Image_11.tif]

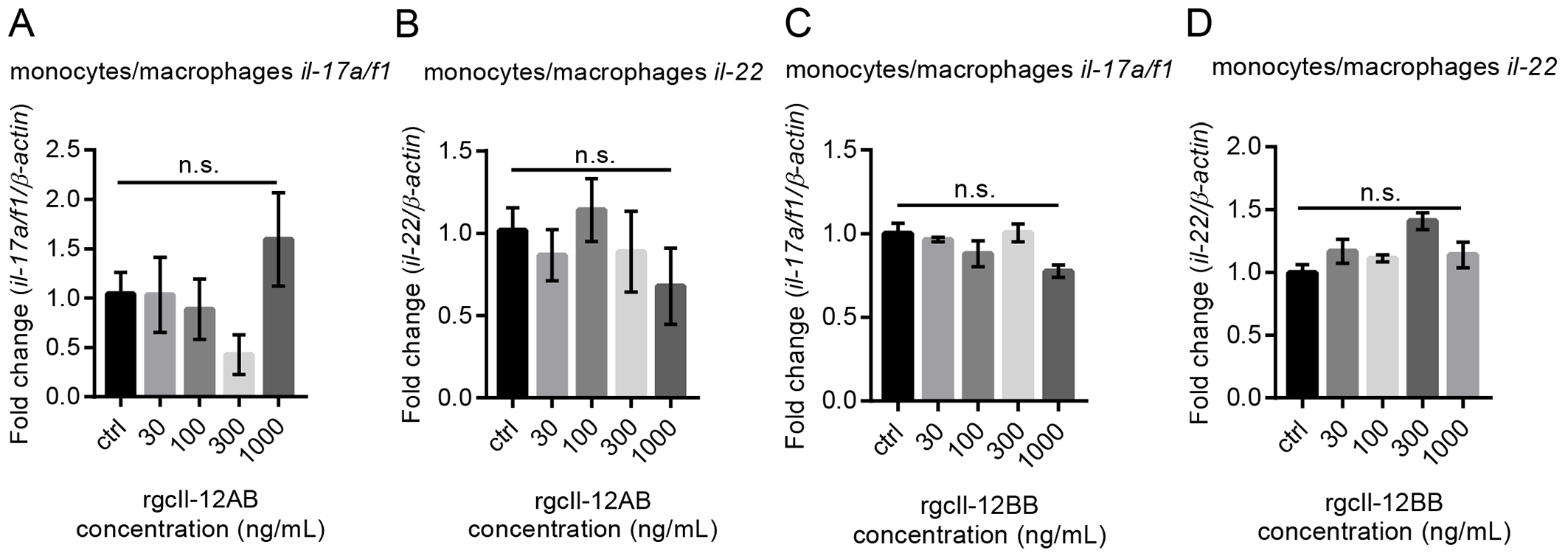

Supplement: Supplementary Figure 12 — Effects of rgcIl-12 isoforms on il-17a/f1 and il-22 mRNA expression in grass carp monocytes/macrophages. Grass carp monocytes/macrophages were treated with 30-1000 ng/mL rgcIl-12AB (A, B) or rgcIl-12BB (C, D) for 12 h. The mRNA expression of il-17a/f1 and il-22 was detected by RT-qPCR and the relative mRNA levels were normalized by β-actin and expressed as fold changes compared with the control group. Data are shown as mean ± SEM (N=4). The “n.s.” indicates no significant. [file Image_12.tif]
