# Supplementary material for: Stimulus-Specific Expression, Selective Generation and Novel Function of Grass Carp (Ctenopharyngodon idella) IL-12 Isoforms: New Insights Into the Heterodimeric Cytokines in Teleosts
Source: Front Immunol. 2021 Sep 16;12:734535. doi: 10.3389/fimmu.2021.734535 (PMC8481787; doi:10.3389/fimmu.2021.734535)
Supplement: Supplementary Table 1 — The primers used in the present study. [file Table_1.pdf]

Supplementary Table 1 Primers used in this study

Primer name	Nucleotide sequence (5'- 3')	Primers information
p35b qcF	AAACACATGGGCAGCGTG	CDS cloning
p35b qcR	AAGCTAAATGACTGCTGGCC	CDS cloning
*p35a-Xho*I(flag) F	CCGCTCGAGATGAAGATCTGCGTTGTG	overexpression
*p35a-EcoR*I(flag) R	GGAATTCCCTTCCTGTGGTCTCCGGAGG	overexpression
*p35b-Xho*I(flag) F	CCGCTCGAGATGCTGCCCAGGGTCTG	overexpression
*p35b-EcoR*I(flag) R	GGAATTCCTTTTGCCAAAATGTAGTTG	overexpression
*p35a-Hind*III G4S3 linker F	AAGCTTGGTGGCGGAGGTTCAGGAGGCGGTGGATCCGGTG GCGGAGGTTCATCTCCGGTGCCTCAGAACACACG	expression
p35a-C-myc+6*his TGA *Xho*I R	CTCGAGTCAATGATGATGATGATGATGGTCGACGGCGCTATT CAGATCCTCTTCTGAGATGAGTTTTTGTTCTAGAAAGCTGGC GGCCGCCTTCCTGTGGTCTCCGGAGGCG	expression
*p35b-Hind*III G4S3 linker F	AAGCTTGGTGGCGGAGGTTCAGGAGGCGGTGGATCCGGTG GCGGAGGTTCAGGTCCTGTCGGAGCGCGTG	expression
*p35b-Xho*I R	CCCAAGCTTGGGATGGACAAGATTGTCTTTTTTATTCT	expression
*p40b-Hind*III F	CATGGACTCGTTGGTGCTGC	expression
*p40b-Hind*III R	AAGCTTTTGTTCCGAGGTGTGCCGG	expression
ifn-γ qF	TGATGACTTTGGGATGGA	real time PCR
ifn-γ qR	AAGACAGGATGTGCGTTG	real time PCR
il-17a/f1 qF	CTCCATGGACCTACACGTTTAC	real time PCR
il-17a/f1 qR	TTGGGTGTAAATAGGTTGGGAC	real time PCR
il-22 qF	GTTCTCCGAGGTCATGTAAAAG	real time PCR
il-22 qR	CTATATCTGTCCTTGTATTTCTTGA	real time PCR
β-actin qF	AGCCATCCTTCTTGGGTATG	real time PCR
β-actin qR	GGTGGGGCGATGATCTTGAT	real time PCR
