# Supplementary material for: Stimulus-Specific Expression, Selective Generation and Novel Function of Grass Carp (Ctenopharyngodon idella) IL-12 Isoforms: New Insights Into the Heterodimeric Cytokines in Teleosts
Source: Front Immunol. 2021 Sep 16;12:734535. doi: 10.3389/fimmu.2021.734535 (PMC8481787; doi:10.3389/fimmu.2021.734535)
Supplement: Supplementary Table 2 — QMEANDisCo Global score of the predicted models. [file Table_2.pdf]

Supplementary Table 2. QMEANDisCo Global score of grass carp Il-12 isoforms structural models.

	gcp35a/p40a	gcp35a/p40b	gcp35a/p40c	gcp35b/p40a	gcp35b/p40b	gcp35b/p40c
QMEANDisCo Global （0~1）	0.58±0.05	0.56±0.05	0.53±0.05	0.60±0.05	0.57±0.05	0.54±0.05
